# Orally Administered Probiotics Decrease *Aggregatibacter actinomycetemcomitans* but Not Other Periodontal Pathogenic Bacteria Counts in the Oral Cavity: A Systematic Review and Meta-Analysis

**DOI:** 10.3389/fphar.2021.682656

**Published:** 2021-08-06

**Authors:** Thanyaporn Sang-Ngoen, László Márk Czumbel, Wuttapon Sadaeng, Alexandra Mikó, Dávid István Németh, Péter Mátrai, Péter Hegyi, Barbara Tóth, Dezső Csupor, István Kiss, Andrea Szabó, Gábor Gerber, Gábor Varga, Beáta Kerémi

**Affiliations:** ^1^ Department of Oral Biology, Semmelweis University, Budapest, Hungary; ^2^ Szentágothai Research Centre, Institute for Translational Medicine, Medical School, University of Pécs, Pécs, Hungary; ^3^ Centre for Translational Medicine, Semmelweis University, Budapest, Hungary; ^4^ Division of Pancreatic Diseases, Heart and Vascular Center, Semmelweis University, Budapest, Hungary; ^5^ Department of Clinical Pharmacy, Faculty of Pharmacy, University of Szeged, Szeged, Hungary; ^6^ Department of Public Health Medicine, Medical School, University of Pécs, Pécs, Hungary; ^7^ Department of Public Health, Faculty of Medicine, University of Szeged, Szeged, Hungary; ^8^ Department of Anatomy, Histology and Embryology, Semmelweis University, Budapest, Hungary

**Keywords:** probiotics, *Aggregatibacter actinomycetemcomitans*, periodontal disease, bacterium, *Porphyromonas gingivalis*, *Tannerella forsythia*, *Prevotella intermedia*, *Fusobacterium nucleatum*

## Abstract

**Introduction:** At the initial part of the gastrointestinal tract, multiple tissues serve the normal function of food delivery. Periodontal structures are integral elements of these. When they deteriorate, it is extremely challenging to regenerate and reconstruct them. The conventional intervention for periodontal disease is scaling and root planning with the aim of reducing pathogenic bacteria. However, periodontal pathogens can rapidly recolonize treated areas. Probiotics have been proposed as novel tools for managing oral health by suppressing pathogenic bacteria through their anti-inflammatory effect, but the available data are controversial.

**Aim:** Therefore, we performed a meta-analysis to study the effect of probiotics on periodontal pathogenic bacteria.

**Methods:** The study was registered in PROSPERO under registration number CRD42018094903. A comprehensive literature search from four electronic databases (PubMed, Cochrane CENTRAL, Embase, and Web of Science) yielded nine eligible records for statistical analysis. Studies measuring bacterial counts in saliva and supra- and subgingival plaque were included. Bacterial counts were analyzed using standard mean difference (SMD) and by a random effects model with the DerSimonian–Laird estimation.

**Results:** The results showed a significant decrease in the overall count of *Aggregatibacter actinomycetemcomitans* in the probiotic-treated group compared to the control at 4 weeks (SMD: −0.28; 95% CI: −0.56–−0.01; *p* = 0.045) but not later. Analyzing the bacterial counts in subgroups, namely, in saliva and supra- and subgingival plaque, separately, yielded no significant difference. Probiotics had no significant effect on the overall count of *Porphyromonas gingivalis* at 4 weeks (SMD: −0.02; 95% CI: −0.35−0.31; *p* = 0.914) or later. Subgroup analysis also revealed no significant difference between treatment and control groups nor did probiotics significantly decrease the overall and subgroup bacterial counts of *Prevotella intermedia*, *Tannerella forsythia*, and *Fusobacterium nucleatum*.

**Conclusion:** Our data support the beneficial effect of probiotics in reducing *A. actinomycetemcomitans* counts, but not of other key periodontal pathogenic bacteria in periodontal disease patients. However, due to the complex mechanism associated with periodontal disease and the limitations of the available studies, there is a further need for well-designed randomized clinical trials to assess the efficacy of probiotics.

## Introduction

In the oral cavity, at the entrance to the gastrointestinal tract, multiple tissues serve the normal function of food delivery. Periodontal structures are integral elements of these. It is extremely challenging to regenerate and reconstruct them when deteriorated. Periodontal disease is a multifactorial, bacteria-induced inflammatory disease of the tooth-supporting structures (Darveau, 2010). Approximately 20–50% of the population are affected worldwide (Nazir, 2017). In susceptible patients, due to bacterial dysbiosis, an uncontrolled and exaggerated inflammatory process develops, which eventually leads to gingival recession, bone resorption, and, consequently, tooth mobility and tooth loss (Windisch et al., 2002; Costalonga and Herzberg, 2014; Hajishengallis, 2014).

The bacteria closely related to periodontal disease are mostly Gram-negative, such as *Porphyromonas gingivalis* (*P. gingivalis*), *Treponema denticola* (*T. denticola*), *Tannerella forsythia* (*T. forsythia*), *Prevotella intermedia* (*P. intermedia*), *Fusobacterium nucleatum* (*F. nucleatum*), and *Aggregatibacter actinomycetemcomitans* (*A. actinomycetemcomitans*) (Roberts and Darveau, 2002; Darveau, 2010; Costalonga and Herzberg, 2014; Hajishengallis, 2014). In addition to the bacteria, host response also plays a key role in the etiology of periodontal disease (Genco, 1992). After bacterial infection, inflammatory mediators are secreted from host immune cells to combat and limit the infected area around the dental tissues (Silva et al., 2015). In addition, smoking, uncontrolled diabetes, and old age, among other important factors, contribute to the inflammation process of the disease (Van Dyke and Sheilesh, 2005). The conventional treatment of periodontal disease includes scaling and root planning as well as the improvement of oral hygiene. These methods target the removal of sub- and supragingival plaque and calculus (Cobb, 2002; Roberts and Darveau, 2002; Claffey et al., 2004). In some cases, because of deep pocket sites in multirooted teeth, scaling and root planning alone are not sufficient, and additional advanced therapies, such as periodontal surgery and the use of antibiotics, are required (Claffey et al., 2004; Cobb, 2008).

Periodontal treatments aim to reduce the number of pathogenic bacteria and remove infected tissue, thereby provoking periodontal tissue healing (Zappa et al., 1991; Cobb, 2002; Claffey et al., 2004). However, periodontopathogens could rapidly recolonize at the previously treated sites even when antibiotics or antiseptics are applied (Cobb, 2002; Quirynen et al., 2005). Thus, scaling and root planning must be performed periodically during the maintenance phase of periodontal disease treatment (Cobb, 2002). Unfortunately, there is insufficient evidence to determine the superiority of different antibiotic protocols (Manresa et al., 2018; Mcgowan et al., 2018). The effectiveness of antibiotic treatments can be very limited, owing to the different antibiotic resistance of the individual species of bacteria and the fact that some bacteria persist intracellularly (Rudney et al., 2005; Teughels et al., 2007; Muniz et al., 2013). Due to these difficulties, there is an increasing need for new treatment modalities to maintain and prolong well-balanced oral microflora and succeed in the therapy of periodontal disease.

Probiotics have been of increasing interest following their success in the treatment of gastrointestinal diseases (Devine and Marsh, 2009). Probiotics are defined as live microorganisms, which, when administered in adequate amounts, confer a health benefit on the host (Sanders, 2008). In dentistry, probiotics have been studied and proposed as a promising alternative treatment to manage oral diseases due to their antimicrobial and anti-inflammatory effects, which may lead to the enrichment of beneficial bacteria (Devine and Marsh, 2009; Allaker and Stephen, 2017). Some clinical trials have reported the favorable effect of probiotics on controlling dental caries, halitosis, and periodontal disease (Burton et al., 2006a; Burton et al., 2006b; Teughels et al., 2013; Vestman et al., 2013; Tekce et al., 2015). On the contrary, other studies have suggested that probiotic treatments do not significantly alter pathogenic flora in the oral cavity (Ahola et al., 2002; Montalto et al., 2004; Montero et al., 2017). Recently published reviews and meta-analyses on this topic have focused on the improvement of clinical periodontal parameters but not on the possible shift in bacterial species in response to probiotics (Yanine et al., 2013; Martin-Cabezas et al., 2016; Akram et al., 2020; Vives-Soler and Chimenos-Küstner, 2020).

Only a few original articles have attempted to study the effect of probiotics on periodontal pathogens in periodontal diseases. Because of the relatively small sample number used and other limitations of these studies, evidence provided is very weak (Gruner et al., 2016; Seminario-Amez et al., 2017; Ikram et al., 2018; Ho et al., 2020). Therefore, in the present meta-analysis, we aimed to study the effect of probiotics on periodontal pathogenic bacteria based on the data from available randomized clinical trials (RCTs).

## Materials and Methods

### Protocol and Registration

This meta-analysis was reported according to the Preferred Reporting Items for Systematic Reviews and Meta-Analyses (PRISMA) statement (Liberati et al., 2009) using the same strategy as in our other recent oral cavity/upper GI-related studies (Czumbel et al., 2019; Keremi et al., 2020; Ruksakiet et al., 2020). The content of this meta-analysis is summarized using the PRISMA checklist (Supplementary Table 1). The study was registered in the international prospective register of systematic reviews, PROSPERO, under the registration number CRD42018094903. There was no deviation from the study protocol.

### Eligibility Criteria

A PICO (patient, intervention, control, and outcome) format was constructed following the clinical question: do orally administered probiotics decrease the quantity of harmful periodontal bacteria in saliva or supra- or subgingival plaque? The PICO framework was the following: patients: periodontal diseases; intervention: orally administered probiotics; control: placebo or no orally administered probiotics. The outcome was the quantity of periodontal pathogenic bacteria in saliva and supragingival and subgingival plaque.

### Inclusion and Exclusion Criteria

Studies that met the following eligibility criteria were included: 1) RCTs, 2) periodontal disease patients, 3) orally administered probiotics, and 4) existing control group. Studies that lacked periodontal pathogenic bacteria counts were excluded. Another exclusion criterion was the application of antibiotics.

### Search Strategy and Information Sources

A systematic search was performed in four electronic databases [MEDLINE (via PubMed), Cochrane Central Register of Controlled Trials (CENTRAL), Embase, and Web of Science] up to June 7, 2020. The electronic search was supplemented by a manual search of bibliographic references from included articles and related review articles. The keyword used for the search was [*probiotic and* (*“periodontal disease” or periodontitis or gingivitis or plaque or saliva*)]. The detailed search string can be found in Supplementary Table 2.

### Study Selection

After duplicate removal, the titles and abstracts in each record were screened by two authors (TS-N and WS) independently. Full texts of the individual records were further assessed by those two authors (TS-N and WS) independently. Disagreements between the reviewers were resolved after discussion or by consulting a third reviewer (GV).

### Data Collection Process and Data Items

Data were collected on the following on predefined data collection spreadsheets: first author, year of publication, number and characteristics of patients, pretreatment, probiotic strain, dose, form, instruction and duration, comparator, and number of periodontal pathogens, such as *A. actinomycetemcomitans*, *P. gingivalis*, *P. intermedia*, *F. nucleatum*, and *T. forsythia* in saliva and supra- and subgingival plaque.

### Risk of Bias Assessment

The Cochrane Risk of Bias Tool for assessing the risk of bias in randomized controlled trials was used. Assessment was performed by two of the authors (TS-N and WS) independently. Disagreements were resolved by consulting a third reviewer (GV). Studies were assessed according to six major domains: random sequence generation (selection bias), allocation concealment (selection bias), blinding of participants and personnel (performance bias), blinding of outcome assessment (detection bias), incomplete outcome data (attrition bias), and selective reporting (reporting bias). Risk of bias in each domain was categorized into low risk, unclear, and high risk (Higgins et al., 2019).

### Summary Measures and Synthesis of Results—Statistical Analysis

Extracted data were pooled using the random effects model with the DerSimonian–Laird estimation and displayed in forest plots as standardized mean difference (SMD) for different methods of measurement. Summary mean estimation, *p* value, and 95% confidence interval (CI) were calculated. *p* < 0.05 was considered as a significant difference from summary mean = 0. Statistical heterogeneity was analyzed using the I^2^ statistic and the chi-square test to ascertain probability values; *p* < 0.1 was defined indicating significant heterogeneity (Higgins et al., 2019).

### Risk of Bias Across Studies and Additional Analyses

The confidence in the body of evidence was graded using the GRADEpro GDT program (McMaster University). Each outcome was assessed following the study design, risk of bias, inconsistency, indirectness, imprecision, and publication bias. A high, moderate, low, or very low grade was assigned to each outcome (Schünemann et al., 2013).

## Results

### Study Selection

**FIGURE 1:**
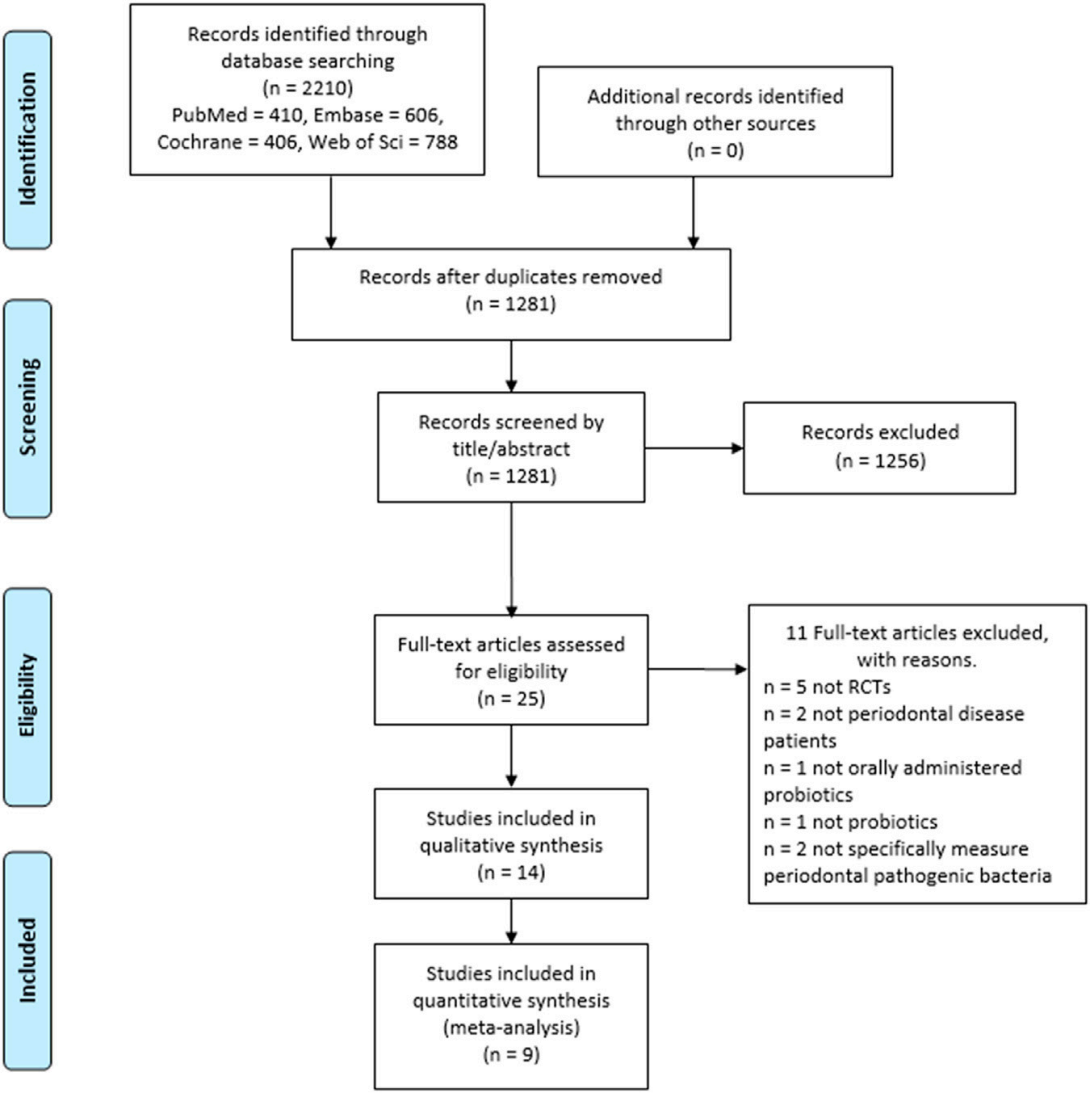
PRISMA flowchart presents the process of study selection.

The comprehensive search from four electronic databases (PubMed, Cochrane Central, Embase, and Web of Science) supplemented by a manual search yielded 1,281 records after duplicate removal. Twenty-five articles were potentially eligible after removing duplicate records and screening by titles and abstracts. After full-text reviews, 14 articles were included in the qualitative analysis, and nine were suitable for the quantitative synthesis (Figure 1).

### Reasons for Exclusions on Full-Text Assessment

In the course of assessing the full-text articles, eleven articles were excluded for good reasons. Out of these, five studies (Zahradnik et al., 2009; Iwamoto et al., 2010; Imran et al., 2015; Kaklamanos et al., 2019; Elsadek et al., 2020) were excluded due to nonrandomized controlled study designs. Six other studies were ineligible owing to their study characteristics. Two articles (Hallstrom et al., 2013; Becirovic et al., 2018) did not investigate periodontal disease patients. Another (Boyeena et al., 2019) used subgingivally delivered probiotics. Tobita and coworkers (Tobita et al., 2018) used killed bacteria, which does not meet the definition of probiotics. Two other studies (Tekce et al., 2015; Swarna Meenakshi and Varghese, 2018) measured total bacterial numbers and obligate anaerobes, which do not fit the purpose of our study. For the quantitative meta-analysis, five studies were excluded for the following reasons. One (Mayanagi et al., 2009) reported periodontal pathogen numbers in graphs which cannot be used in our statistical method. An email requesting the exact number of bacteria was sent to the corresponding author of the study; however, no reply was received. One study (Goyal et al., 2019) did not specify whether the participants had periodontal disease, and another (Vivekananda et al., 2010) waited 21 days before administering probiotics to the participants. Finally, two other investigations (Shah et al., 2013; Shah et al., 2017) combined probiotics with antibiotics.

### Characteristics of the Studies Included

Eight of the 14 studies (Mayanagi et al., 2009; Teughels et al., 2013; Laleman et al., 2015; Montero et al., 2017; Alanzi et al., 2018; Invernici et al., 2018; Morales et al., 2018; Laleman et al., 2019) were randomized, double-blind, placebo-controlled parallel trials. The other four investigations (Shah et al., 2013; Dhaliwal et al., 2017; Shah et al., 2017; Goyal et al., 2019) were randomized, open, controlled parallel trials. The work of Iniesta and coworkers (Iniesta et al., 2012) was a randomized, double-blind, placebo-controlled, crossover clinical trial. An additional one (Vivekananda et al., 2010) had a randomized, placebo-controlled, double-blind, split-mouth design. The age of participants varied from adolescent to elderly.

Different probiotic strains and doses are used for intervention (Supplementary Table 3). Commonly used forms of probiotics are tablets or lozenges. However, other formulations, such as mouthwash or a sachet, are also in use. Instructions for use depended on the probiotic products (Supplementary Table 3). The duration of use ranged from 4 weeks to 3 months. In most studies, the investigated periodontal pathogenic bacteria were *A. actinomycetemcomitans*, *P. gingivalis*, *P. intermedia*, *T. forsythia*, and *F. nucleatum*.

### Risk of Bias Assessment

**FIGURE 2:**
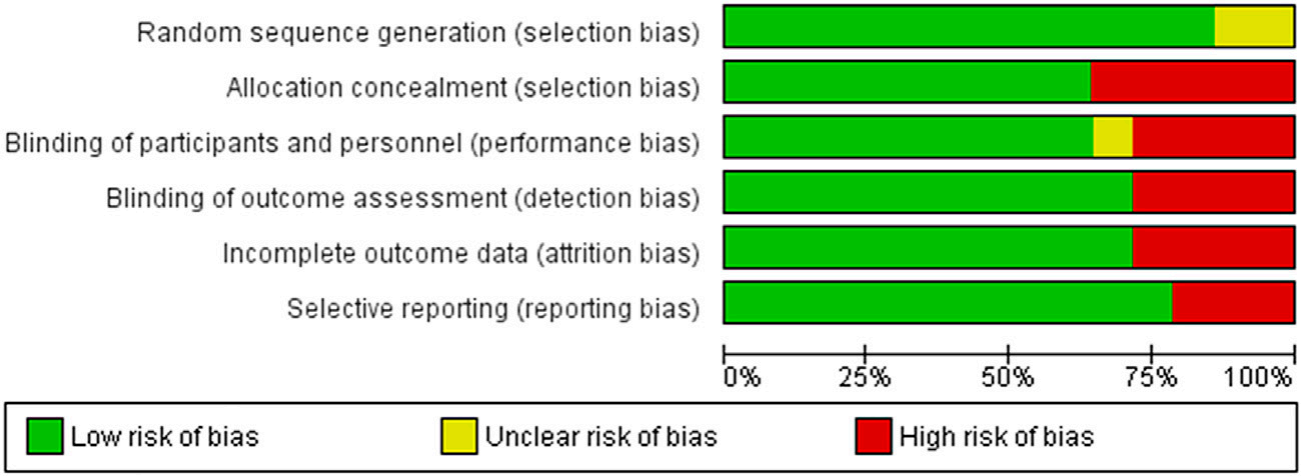
Risk of bias graph presented each risk of bias item as percentages across all included studies.

Biases in the 14 included studies were assessed by using the Cochrane Risk of Bias Tool for randomized trials. All included studies identified or explained the randomization method, except one (Goyal et al., 2019), which did not specify the method. Allocation concealment was determined as high in five studies because the staff who assigned the participants to the groups was not blinded. Most of the included studies had a double-blind design, and the performance and detection biases were evaluated as low. However, four studies (Shah et al., 2013; Dhaliwal et al., 2017; Shah et al., 2017; Goyal et al., 2019) were open trials; the performance and detection biases of these were therefore determined as high. One study (Morales et al., 2018) was assessed as having an unclear risk in performance bias for microbiological parameters because the sample collector was not blinded. Four studies (Dhaliwal et al., 2017; Montero et al., 2017; Morales et al., 2018; Goyal et al., 2019) incompletely reported microbiological data with no explanation. High reporting bias was found in three studies (Laleman et al., 2015; Montero et al., 2017; Morales et al., 2018) because they did not report all prespecified outcomes. The result of the risk of bias assessment can be found in Figure 2 and Supplementary Figure 1, and the details of assessment in each included study can be found in Supplementary Table 4.

### Results of the Meta-Analysis

**FIGURE 3:**
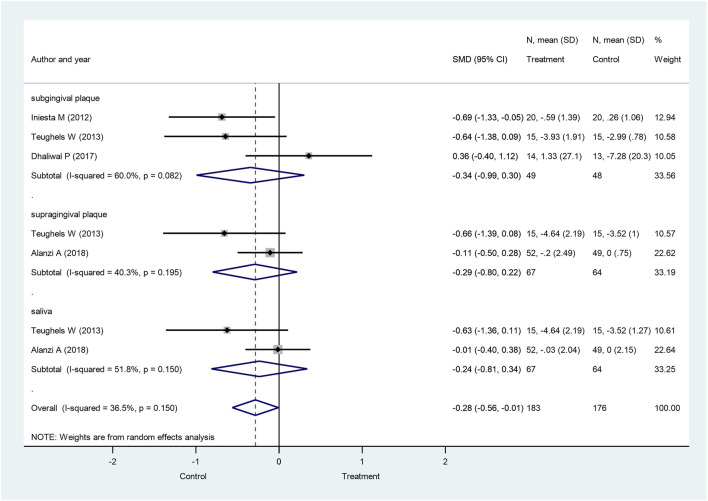
Forest plot analysis of the change in *Aggregatibacter actinomycetemcomitans* at 4 weeks. The overall result presented a significant decrease of *Aggregatibacter actinomycetemcomitans* in the treatment group compared to the control group (SMD: −0.28; 95% CI: −0.56–−0.01; *p* = 0.045).

Out of the five investigated periodontal pathogenic bacteria, *A. actinomycetemcomitans* exhibited the greatest response to probiotics treatment. When subgingival and supragingival changes were examined together with salivary bacteria counts, the overall *A. actinomycetemcomitans* bacteria counts were significantly lower in the probiotic-treated group than in the control at 4 weeks (SMD: −0.28; 95% CI: −0.56–−0.01; *p* = 0.045). There were no significant heterogeneity differences between the studies (I^2^ = 36.5%; *p* = 0.150). However, the subgroup analysis revealed no difference in *A. actinomycetemcomitans* between the probiotics group and the control (Figure 3). *A. actinomycetemcomitans* values in subgingival plaque taken from three studies (Iniesta et al., 2012; Teughels et al., 2013; Dhaliwal et al., 2017) involving a total of 97 participants (49 subjects in the treated group and 48 people in the control) showed no significant difference between the probiotics group and the control group (SMD: −0.34; 95% CI: −0.99–0.30; *p* = 0.297), but, in this respect, significant heterogeneity was found among the studies (I^2^ = 60%, *p* = 0.082). As regards supragingival plaque and saliva, two studies (Teughels et al., 2013; Alanzi et al., 2018) involving 131 participants (67 probiotic-treated and 64 control subjects) showed no significant difference between the two groups either (SMD: −0.29; 95% CI: −0.80–0.22; *p* = 0.262; and SMD: −0.24; 95% CI: −0.81–0.34; *p* = 0.420, respectively). Significant heterogeneity among the studies was found again for both supragingival plaque and saliva values (I^2^ = 40.3%, *p* = 0.195; I^2^ = 51.8%, *p* = 0.150, respectively).

**FIGURE 4:**
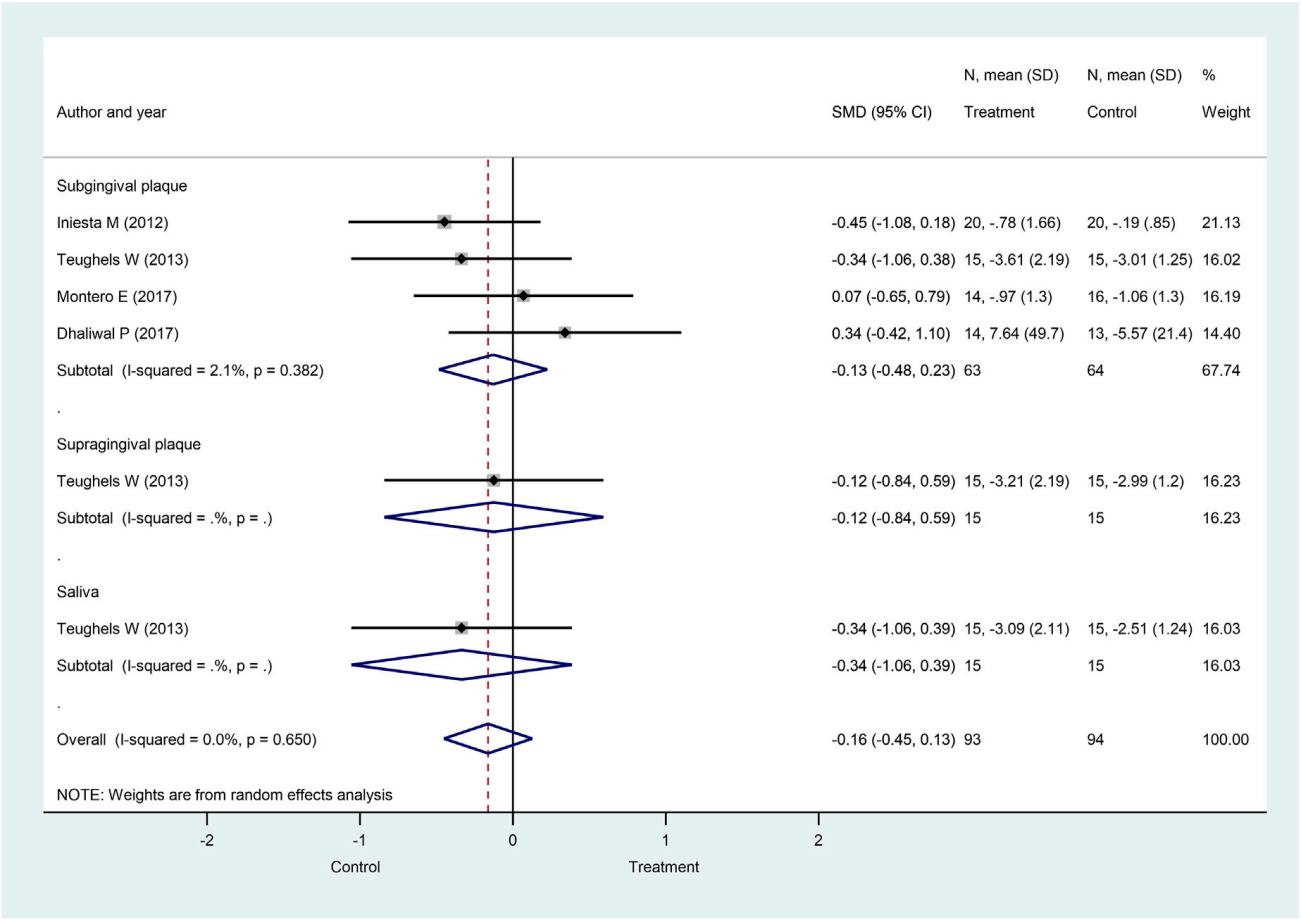
Forest plot analysis of the change in *Aggregatibacter actinomycetemcomitans* at 8 weeks. The overall result presented no significant difference of bacterial decrease when both treatment and control groups were compared (SMD: −0.16; 95% CI: −0.45–0.13; *p* = 0.271).

Eight weeks after the initiation of probiotics treatment, when subgingival, supragingival, and saliva bacteria counts were combined, the overall *A. actinomycetemcomitans* bacteria counts showed a tendency to decrease, but this difference fell short of significance between the probiotic-treated and the control groups (SMD: −0.16; 95% CI: −0.45–0.13; *p* = 0.271) with no significant heterogeneity (I^2^ = 0.0%, *p* = 0.650). Similarly, the subgroup analysis showed no significant difference in subgingival plaque (SMD: −0.13; 95% CI: −0.48–0.23; *p* = 0.474) and no significant heterogeneity (I^2^ = 2.1%; *p* = 0.382) in supragingival plaque (SMD: −0.12; 95% CI: −0.84–0.59; *p* = 0.733) and in saliva (SMD: −0.34; 95% CI: −1.06–0.39; *p* = 0.362) (Figure 4).

**FIGURE 5:**
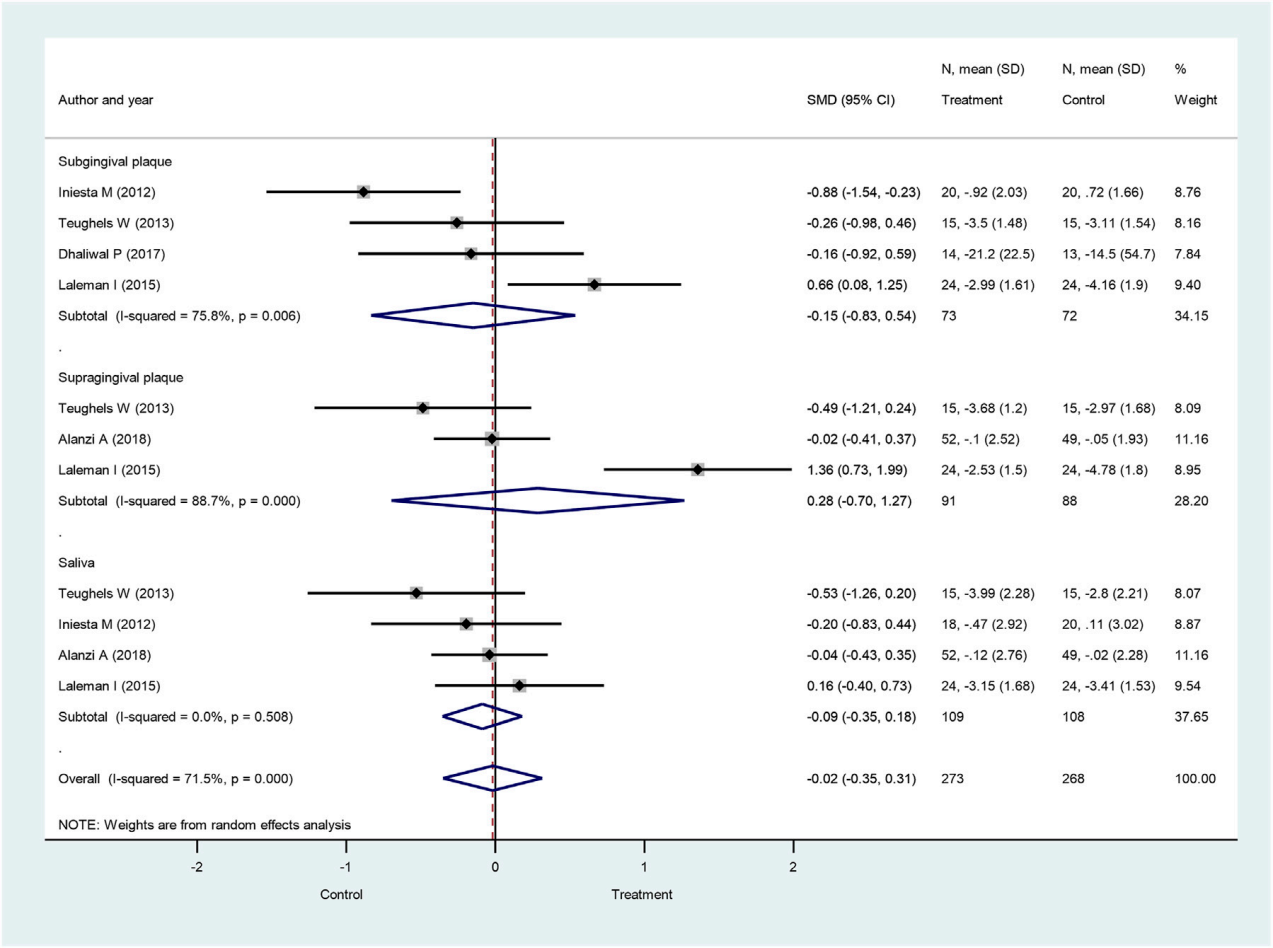
Forest plot analysis of the change in *Porphyromonas gingivalis* at 4 weeks. The overall result presented no significant difference of bacterial decrease when both treatment and control groups were compared (SMD: −0.02; 95% CI: −0.35–0.31; *p* = 0.914).

Another pathogenic bacterium regarded as a key factor in the development of periodontitis is *P. gingivalis*. When subgingival and supragingival changes were combined with salivary bacteria counts, the overall *P. gingivalis* bacteria counts were not significantly different between the probiotic-treated and the control groups at 4 weeks (SMD: −0.02; 95% CI: −0.35–0.31; *p* = 0.914) (Figure 5), 8 weeks (SMD: −0.01; 95% CI: −0.53–0.52; *p* = 0.977) (Supplementary Figure 2), and 12 weeks after treatment initiation (SMD: −0.23; 95% CI: −0.90–0.43; *p* = 0.488) (Supplementary Figure 3).

When only subgingival plaque *P. gingivalis* values were involved, again, no significant differences were observed between the probiotic-treated group and the control at 4 weeks (SMD: −0.15; 95% CI: −0.83–0.54; *p* = 0.670) (Figure 5), 8 weeks (SMD: −0.04; 95% CI: −0.61–0.52; *p* = 0.877) (Supplementary Figure 2), and 12 weeks after treatment initiation (SMD: −0.08; 95% CI: −1.15–0.98; *p* = 0.876) (Supplementary Figure 3). We also analyzed *P. gingivalis* counts in supragingival plaque. Similar to the findings above, the bacteria counts were not significantly different between the probiotic-treated and the control group at 4 weeks (SMD: 0.28; 95% CI: −0.70–1.27; *p* = 0.570) (Figure 5), 8 weeks (SMD: 0.41; 95% CI: −2.43–3.25; *p* = 0.779) (Supplementary Figure 2), and 12 weeks after treatment initiation (SMD: −0.21; 95% CI: −1.96–1.54; *p* = 0.813) (Supplementary Figure 3). Similar results were found for *P. gingivalis* bacteria numbers in saliva, yielding no significant differences between the probiotic-treated and the control groups at 4 weeks (SMD: −0.09; 95% CI: −0.35–0.18; *p* = 0.519) (Figure 5), 8 weeks (SMD: −0.20; 95% CI: −0.87–0.47; *p* = 0.564) (Supplementary Figure 2), and 12 weeks after treatment initiation (SMD: −0.42; 95% CI: −1.52–0.68; *p* = 0.455) (Supplementary Figure 3).

**FIGURE 6:**
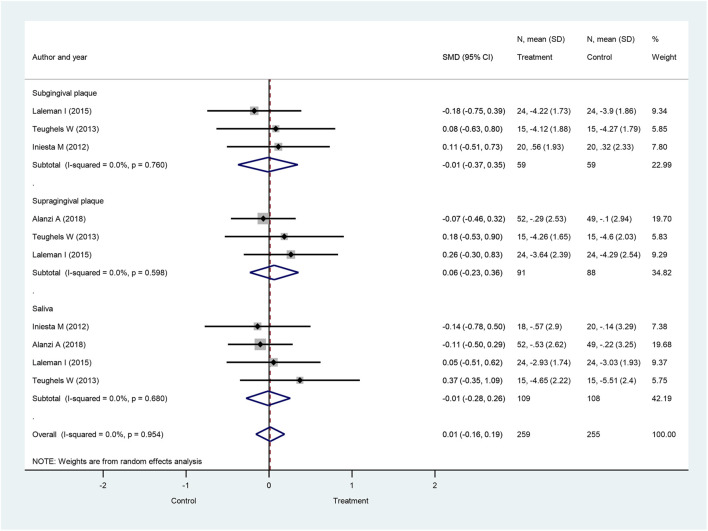
Forest plot analysis of the change in *Prevotella intermedia* at 4 weeks. The overall result presented no significant difference of bacterial decrease when both treatment and control groups were compared (SMD: 0.01; 95% CI: −0.16–0.19; *p* = 0.874).

We also studied the bacteria count changes investigating three additional pathogenic bacteria, *P. intermedia*, *F. nucleatum*, and *T. forsythia*, which are also assumed to play a crucial role in the course of periodontitis development. When subgingival, supragingival, and salivary bacteria counts were combined, the overall *P. intermedia* bacteria counts did not significantly differ between the probiotic-treated group and the control group at 4 weeks (SMD: 0.01; 95% CI: −0.16–0.19; *p* = 0.874) (Figure 6), 8 weeks (SMD: −0.01; 95% CI: −0.24–0.21, *p* = 0.912) (Supplementary Figure 4), and 12 weeks after treatment initiation (SMD: −0.00; 95% CI: −0.30–0.29; *p* = 0.981) (Supplementary Figure 5). Similar observations were made in subgroup analysis, when subgingival, supragingival, and salivary *P. intermedia* bacteria counts were investigated separately, showing no significant differences between the treatment groups.

**FIGURE 7:**
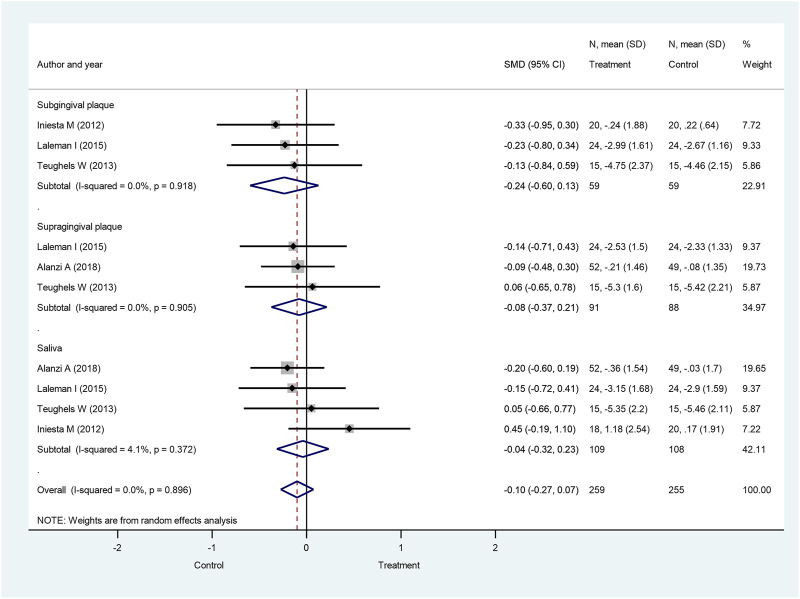
Forest plot analysis of the change in *Fusobacterium nucleatum* at 4 weeks. The overall result presented no significant difference of bacterial decrease when both treatment and control groups were compared (SMD: −0.10; 95% CI: −0.27–0.07; *p* = 0.256).

For *F. nucleatum*, combined subgingival, supragingival, and salivary bacteria counts yielded no significantly different results between the probiotic-treated group and the control group at 4 weeks (SMD: −0.10; 95% CI: −0.27–0.07; *p* = 0.256) (Figure 7), 8 weeks (SMD: 0.12; 95% CI: −0.09–0.32; *p* = 0.270) (Supplementary Figure 6), and 12 weeks after treatment initiation (SMD: −0.06; 95% CI: −0.27–0.15; *p* = 0.580) (Supplementary Figure 7). When subgingival, supragingival, and salivary count changes were investigated separately, subgroup analysis also demonstrated the lack of significant differences between treatment groups.

**FIGURE 8:**
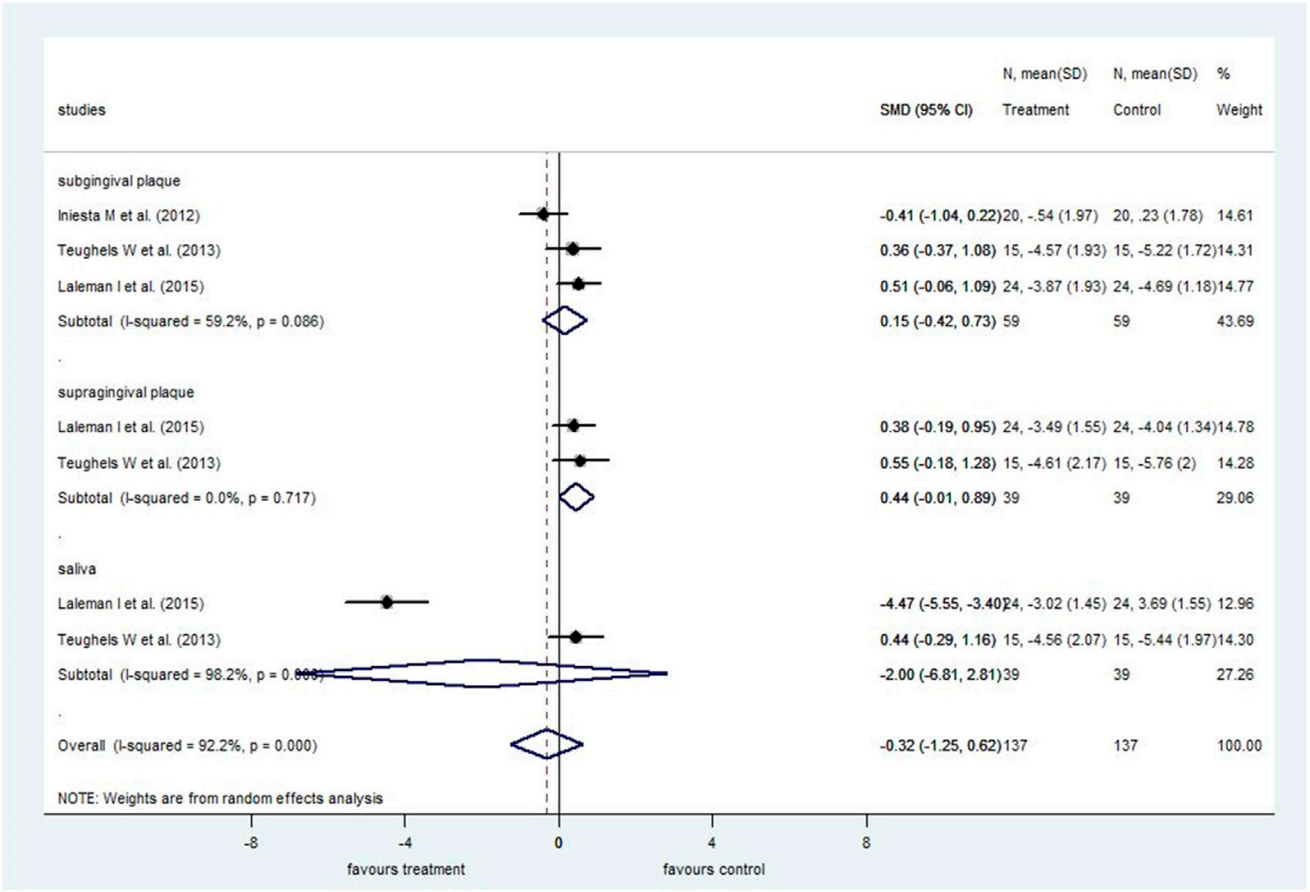
Forest plot analysis of the change in *Tannerella forsythia* at 4 weeks. The overall result presented no significant difference of bacterial decrease when both treatment and control groups were compared (SMD: −0.32; 95% CI: −1.25–0.62).

Finally, when subgingival and supragingival counts were combined with salivary bacteria counts, the overall *T. forsythia* bacteria counts were not significantly different between the probiotic-treated group and the control group at 4 weeks (SMD: −0.32; 95% CI: −1.25–0.62) (Figure 8) and 8 weeks (SMD: 0.05; 95% CI: −0.19–0.30) (Supplementary Figure 8). Similarly, no significant differences were found between the treatment groups using subgroup analysis, investigating subgingival, supragingival, and salivary counts separately.

### The Quality of Evidence

The GRADE assessment of periodontal pathogen outcomes was very low due to serious risk bias, inconsistency, and imprecision (Table 1). The details of grading the results can be seen in Supplementary Table 5. According to the certainty classification of the GRADE system, the low level indicates that further research is very likely to have an important impact on our confidence in estimating effect and is likely to change the estimate. The very low level of certainty suggests that any estimate of effect is very uncertain.

**TABLE 1:** Summary of **s**tatistical analysis for the microbiological outcomes.

Outcomes	No. of studies in meta-analysis	Patients (n)	Results		Heterogeneity		Quality
			Meta-analysis overall estimate (95%CI)	*p*	I^2^ (%)	*p*	
*A. actinomycetemcomitans* at 4 weeks	4	198	SMD: −0.28 (−0.56, −0.01)	0.045	36.5	0.150	⊕○○○
*A. actinomycetemcomitans* at 8 weeks	4	127	SMD: −0.16 (−0.45, 0.13)	0.271	0.00	0.650	⊕○○○
*P. gingivalis* at 4 weeks	5	246	SMD: −0.02 (−0.35, 0.31)	0.914	71.5	0.000	⊕○○○
*P. gingivalis* at 8 weeks	5	196	SMD: −0.01 (−0.53, 0.52)	0.977	84.3	0.000	⊕○○○
*P. gingivalis* at 12 weeks	3	117	SMD: −0.23 (−0.90, 0.43)	0.488	88.7	0.000	⊕○○○
*P. intermedia* at 4 weeks	4	219	SMD: 0.01 (−0.16, 0.19)	0.874	0.00	0.954	⊕○○○
*P. intermedia* at 8 weeks	3	118	SMD: −0.01 (−0.24, 0.21)	0.912	0.00	0.814	⊕○○○
*P. intermedia* at 12 weeks	4	144	SMD: −0.00 (−0.30, 0.29)	0.981	51.3	0.030	⊕○○○
*F. nucleatum* at 4 weeks	5	267	SMD: −0.10 (−0.27, 0.07)	0.256	0.00	0.896	⊕○○○
*F. nucleatum* at 8 weeks	4	170	SMD: 0.12 (−0.09, 0.32)	0.270	0.00	0.791	⊕○○○
*F. nucleatum* at 12 weeks	3	117	SMD: −0.06 (−0.27, 0.15)	0.580	0.00	0.933	⊕○○○
*T. forsythia* at 4 weeks	3	128	SMD: −0.32 (−1.25, 0.62)	0.851	92.2	0.000	⊕○○○
*T. forsythia* at 8 weeks	4	167	SMD: 0.05 (−0.19, 0.30)	0.685	18.8	0.281	⊕○○○

## Discussion

### Summary of Evidence

In the present work, we conducted a meta-analysis comparing periodontal pathogenic bacteria counts between probiotic-treated and placebo-treated control groups of patients suffering from periodontal diseases. In our analysis, the primary focus was to study the effect of probiotics by evaluating the colony-forming unit (CFU) counts of pathogenic bacteria. Five different species at three anatomical sites (saliva, supragingival, and subgingival areas) were analyzed. To strengthen the grade of evidence, only RCTs were included in our study. We found a significant decrease in *A. actinomycetemcomitans* counts in the probiotic-treated group compared to the control group at 4 weeks after treatment initiation, but the difference fell short of significance after 8 weeks. There were no significant differences in the other four periodontal pathogenic bacteria, *P. gingivalis, P. intermedia, F. nucleatum*, and *T. forsythia*, at any time points when counts were compared between the two groups.

Up until now, three systematic reviews and one meta-analysis have investigated the effect of probiotics on periodontal pathogens. Among these, two published articles claimed that it was impossible to draw definite conclusions about the effectiveness of probiotics due to the limited number of available studies (Seminario-Amez et al., 2017; Ikram et al., 2018). One systematic review did not specify the investigated periodontal pathogens but claimed that probiotics do not cause a diminishing effect on periodontal pathogens (Seminario-Amez et al., 2017). Only one meta-analysis (Gruner et al., 2016) used only two RCT studies for meta-analysis, which are obviously not sufficient to perform an analysis with acceptable statistical power. That work reported the diminishing effect of probiotics on *A. actinomycetemcomitans* and no effect on *P. gingivalis* and *P. intermedia*. Although these results can only be regarded as qualitative, they clearly show similarities to our data, which were drawn from far more studies and therefore much higher sample numbers. However, there are some differences between their work and our studies. Gruner and coinvestigators (Gruner et al., 2016) in the two included studies used bacteria numbers which were only obtained at the latest follow-up time points, combining eight- and twelve-week results. In contrast, we included more studies, more samples, and more time points. Another article also reported an analysis which was only based on two original articles (Ho et al., 2020), also reporting no significant effects on *P. gingivalis*, *P. intermedia*, *F. nucleatum*, and *T. forsythia* in response to probiotic treatment.

Our study included all types of periodontal diseases in our meta-analysis in order to primarily focus on the change in periodontal pathogen counts during periodontal diseases. Additionally, we included studies using different strains of probiotics to yield conclusive results. Among these included studies, four (Vivekananda et al., 2010; Iniesta et al., 2012; Teughels et al., 2013; Laleman et al., 2019) used *L. reuteri*. Two studies (Alanzi et al., 2018; Goyal et al., 2019) employed a mixture of *L. reuteri* with other beneficial bacteria. The remaining RCTs used other probiotic strains. The most commonly used probiotic, *L. reuteri*, was reported to show a potency to overcome pathogenic microorganisms because of its antimicrobial compounds, reuterin and reutericyclin, and it was also described as having an immunomodulatory effect on the host (Britton, 2017).

Some clinical trials included in our article investigated the antibacterial effects of *L. reuteri* and showed a reduction in the number of periodontal pathogens, such as *A. actinomycetemcomitans*, *P. gingivalis*, and *T. forsythia*, in patients with periodontal diseases (Vivekananda et al., 2010; Iniesta et al., 2012; Teughels et al., 2013; Goyal et al., 2019). Additionally, this antimicrobial effect is supported by three *in vitro* experiments (Kang et al., 2011; Baca-Castan et al., 2014; Santos et al., 2020). One included study (Goyal et al., 2019) used a mixture of *L. reuteri*, *L. rhamnosus*, *B. longum*, and *B. bifidum* and showed the beneficial effect at 24 weeks, while another included study (Laleman et al., 2019) showed no beneficial effect of probiotics on periodontal pathogens at 12 and 24 weeks. This might suggest that long-term use of *L. reuteri* can limit the antibacterial effect, and a mixture of *L. reuteri* was possibly used in the long term to prolong the antibacterial effect. The dose of *L. reuteri* used in three trials (Vivekananda et al., 2010; Iniesta et al., 2012; Teughels et al., 2013) was at least 2 × 10^8^ CFU per day, which seems to be an effective dose.

Three previous meta-analyses reported a specific effect of *L. reuteri* on clinical periodontal parameters (Martin-Cabezas et al., 2016; Ikram et al., 2018; Akram et al., 2020). Martin-Cabesaz and coinvestigators (Martin-Cabezas et al., 2016) reported beneficial effects of probiotics on improving CAL gain and reducing PPD in both moderate and deep pockets. Moreover, they described a significant reduction of BOP in the probiotic group in the short term (combining 6- and 12-week results). Another meta-analysis (Ikram et al., 2018) suggested the effectiveness of probiotics on CAL gain in chronic periodontitis compared to controls; however, they were not able to demonstrate this for PPD reduction. Another meta-analysis (Akram et al., 2020) investigated the effect of *L. reuteri* in gingivitis patients and suggested no statistically significant difference in GI and PI between the probiotic-treated and control groups. These three meta-analyses of periodontal diseases report quite diverse responses to *L. reuteri* treatment.

Another previous meta-analysis (Ho et al., 2020) reported significant CAL gain and PPD reduction in chronic periodontitis at 3 months. Additionally, published reviews indicated variable results, either supporting or questioning the effectiveness of probiotics on the clinical parameters of periodontal diseases (Deepa and Mehta, 2009; Yanine et al., 2013; Matsubara et al., 2016; Seminario-Amez et al., 2017; Barboza et al., 2020; Vives-Soler and Chimenos-Küstner, 2020). The reason for the controversial results may depend on a number of factors, including the pooling of very different follow-up times of the included RCTs, the use of a wide variety of probiotics and their dosage in the included RCTs, and variations in patient characteristics.

The efficacy of probiotics probably relies on the actual bacteria strain, dose, and follow-up time, as described in the management of gastrointestinal tract disorders (Verna and Lucak, 2010; Ciorba, 2012; Kechagia et al., 2013). Various probiotic species may have divergent effects on pathogenic bacteria (Schrezenmeir and de Vrese, 2001; Verna and Lucak, 2010). In addition to the bacteria strain of probiotics, the dose of consumption is also important. The minimum effective doses of probiotics are still controversial; however, it is generally accepted that probiotic products should be consumed daily for a total of 10^8^—10^9^ probiotic microorganisms (Ciorba, 2012). Additionally, the characteristics of patients should also be considered as different gastrointestinal tract diseases caused by different pathogenic bacteria could be cured by different probiotic strains (Verna and Lucak, 2010). The occurrence of different pathogenic bacteria related to each type of periodontal diseases may also be different (Ardila et al., 2012; Farias et al., 2012). Thus, the type of periodontal disease could be important for the selection of probiotics in future studies.

The safety of probiotics is also important. Three studies (Mayanagi et al., 2009; Vivekananda et al., 2010; Dhaliwal et al., 2017) reported no adverse effects of probiotics during the trial, while two studies (Iniesta et al., 2012; Montero et al., 2017) described abdominal pain resulting from increased intestinal motility, which can be considered as a mild side effect. Another study (Laleman et al., 2019) reported altered sensations in the oral cavity.

Considering our findings on microbiological data, *A. actinomycetemcomitans* has been shown to induce bone loss, periodontal pocket formation, and clinical attachment loss during periodontitis (Mooney and Kinane, 1994; Fine et al., 2007). Furthermore, successful periodontal treatment is often based on the reduction of depth of the periodontal pocket (Donos, 2018). Therefore, our results could suggest that probiotics decrease *A. actinomycetemcomitans*, allowing the healing of tooth-supporting tissues and resulting in clinical parameters in cases when the high count of this bacterium plays a significant pathological role in the course of periodontal disease.

### Limitations

The present meta-analysis provides evidence-based answers through appropriately selected articles and processed data. Bacteria numbers on a continuous scale were used for statistical analysis; the change in the bacteria number thus clearly demonstrated, analyzed, and accurately indicated the results. Nevertheless, some unavoidable limitations are present in our study. The major limitation is the low number of included trials and the relatively high heterogeneity. Only nine articles were included in the statistical analysis. These articles used different probiotic strains; a clear outcome for specific probiotic strains could therefore not be produced. The heterogeneity of the included studies, for example, different probiotic strains, doses, and forms, was ignored to yield sufficient data for statistical analysis. We diminished these differences by using means and standard deviations for result synthesis. However, some hidden confounding factors could have affected the results, such as the microbial culture count, subjective decision on the plaque index and gingival index, and the probing pressure used. Furthermore, according to Matsubara et al. (2016), each probiotic strain may have a different effect on each pathogenic species. Furthermore, studies were conducted in different regions of the world, thus creating diverse environmental factors. The different genetic and genomic background of the patients may also have interfered with the efficacy of probiotic strains and have had an overall altering effect on treatment. Additionally, oral hygienic instructions and cleaning of teeth or scaling and root planning could vary across studies, which may also have led to the heterogeneity of the results.

In conclusion, based on the included data, orally administered probiotics decrease *A. actinomycetemcomitans* counts. In contrast, no beneficial effect of probiotics was observed for the other investigated periodontal pathogens. Our study highlights the heterogeneity among the available RCTs and the need for standardized clinical protocols in the future to evaluate the effect of various probiotics on periodontal pathogens.
