# Supplementary figures and images for: Orally Administered Probiotics Decrease Aggregatibacter actinomycetemcomitans but Not Other Periodontal Pathogenic Bacteria Counts in the Oral Cavity: A Systematic Review and Meta-Analysis
Source: Front Pharmacol. 2021 Aug 6;12:682656. doi: 10.3389/fphar.2021.682656 (PMC8383782; doi:10.3389/fphar.2021.682656)

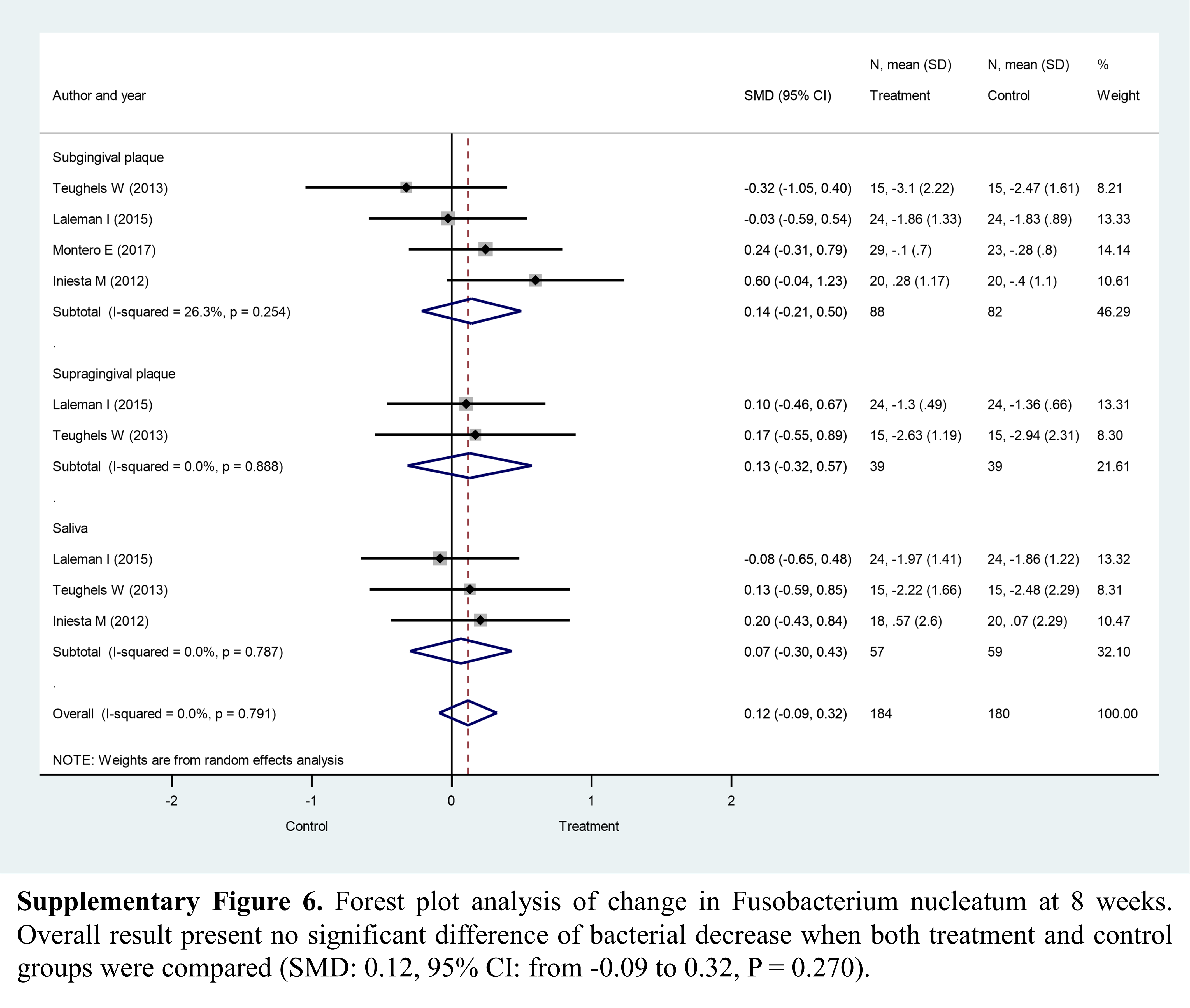

Supplement: Supplementary file 1 [file Image6.TIF]

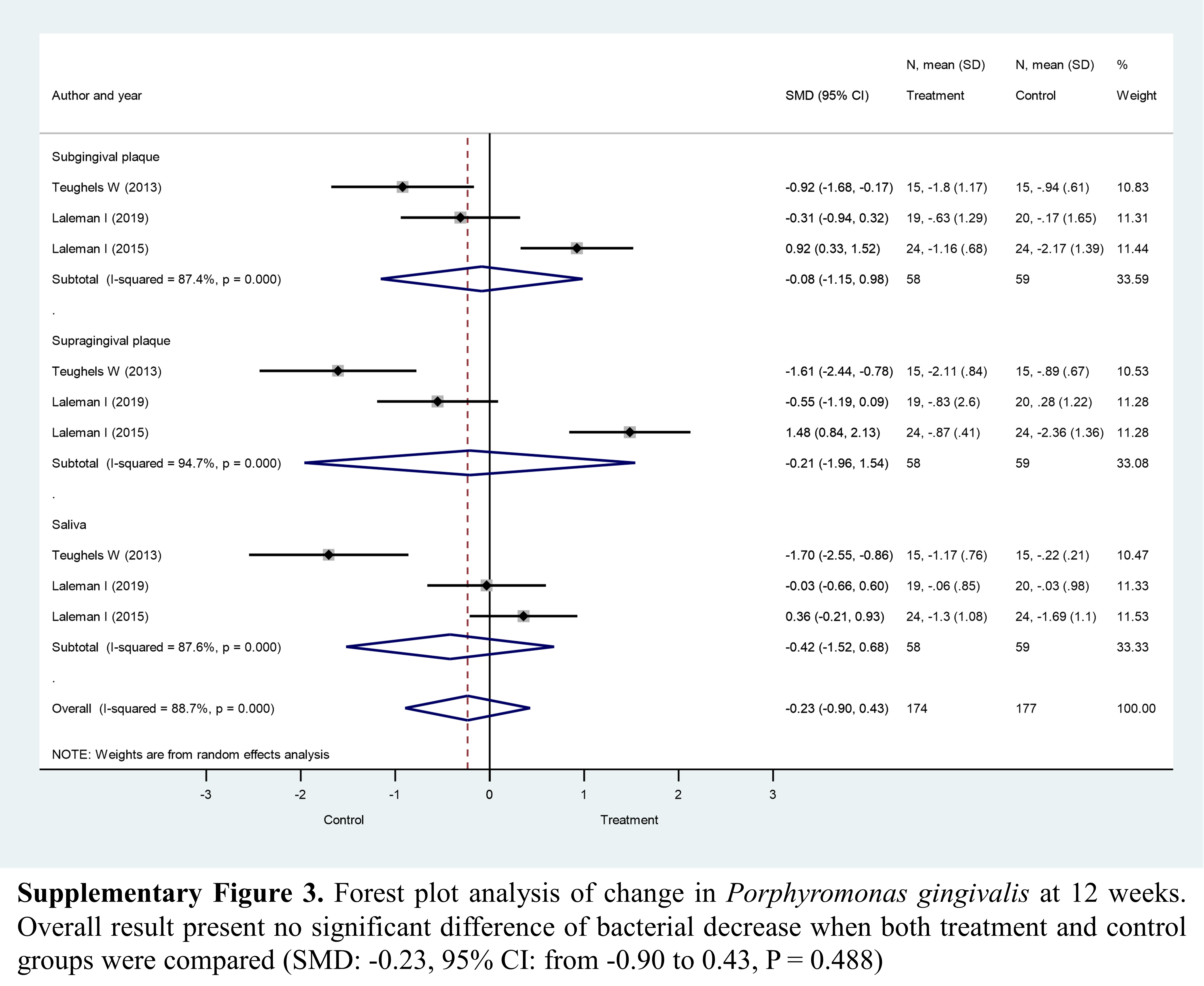

Supplement: Supplementary file 3 [file Image3.TIF]

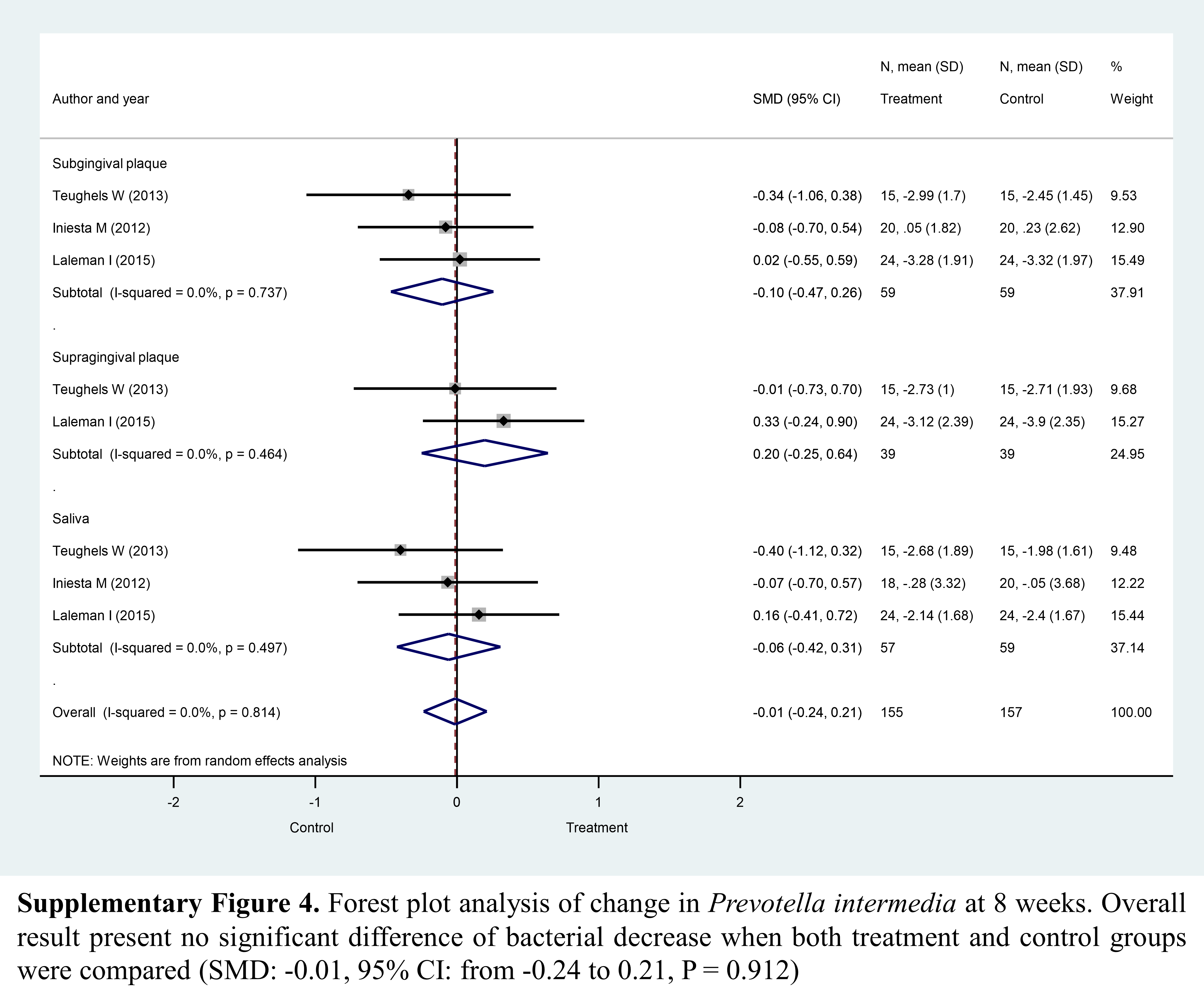

Supplement: Supplementary file 4 [file Image4.TIF]

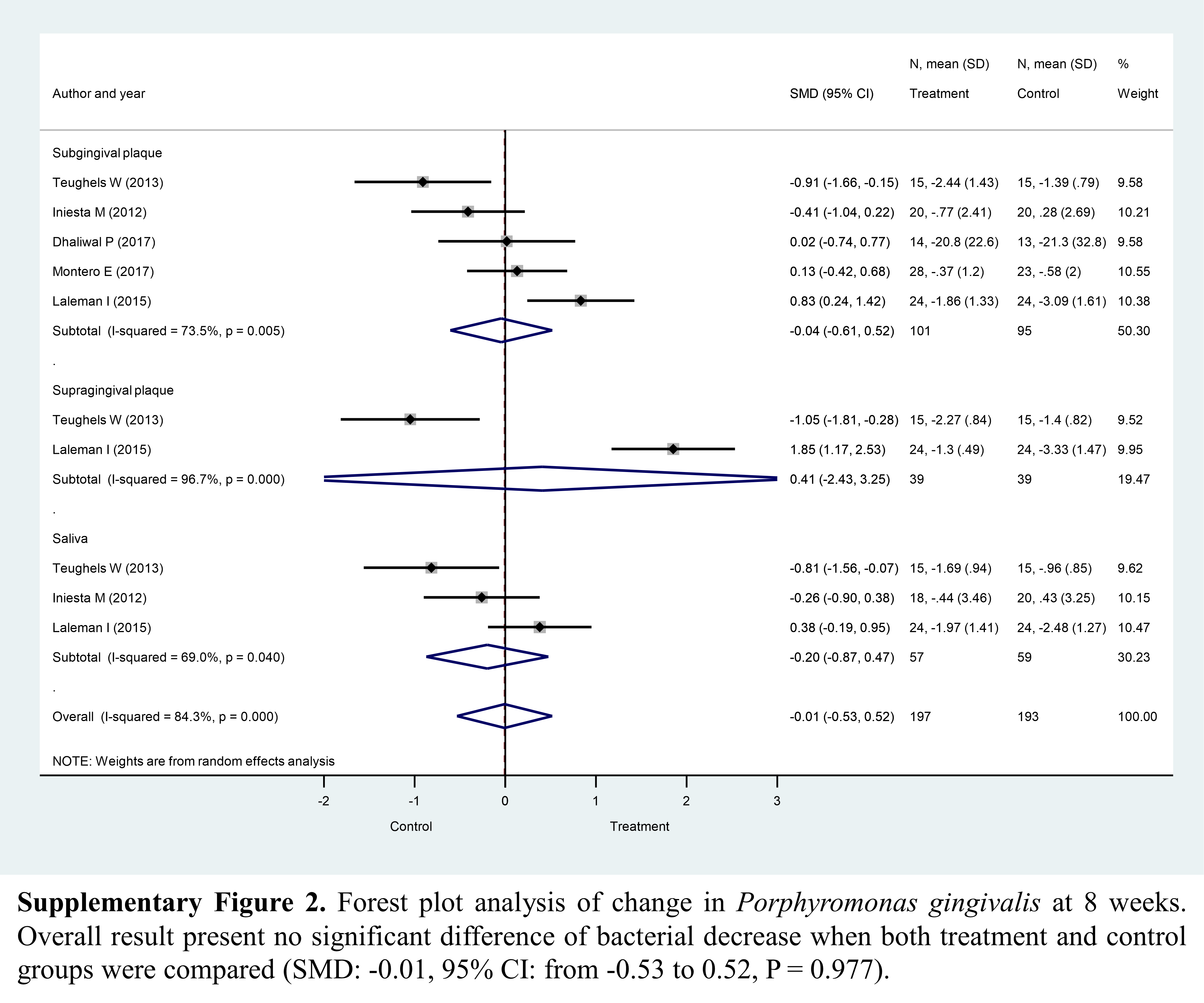

Supplement: Supplementary file 5 [file Image2.TIF]

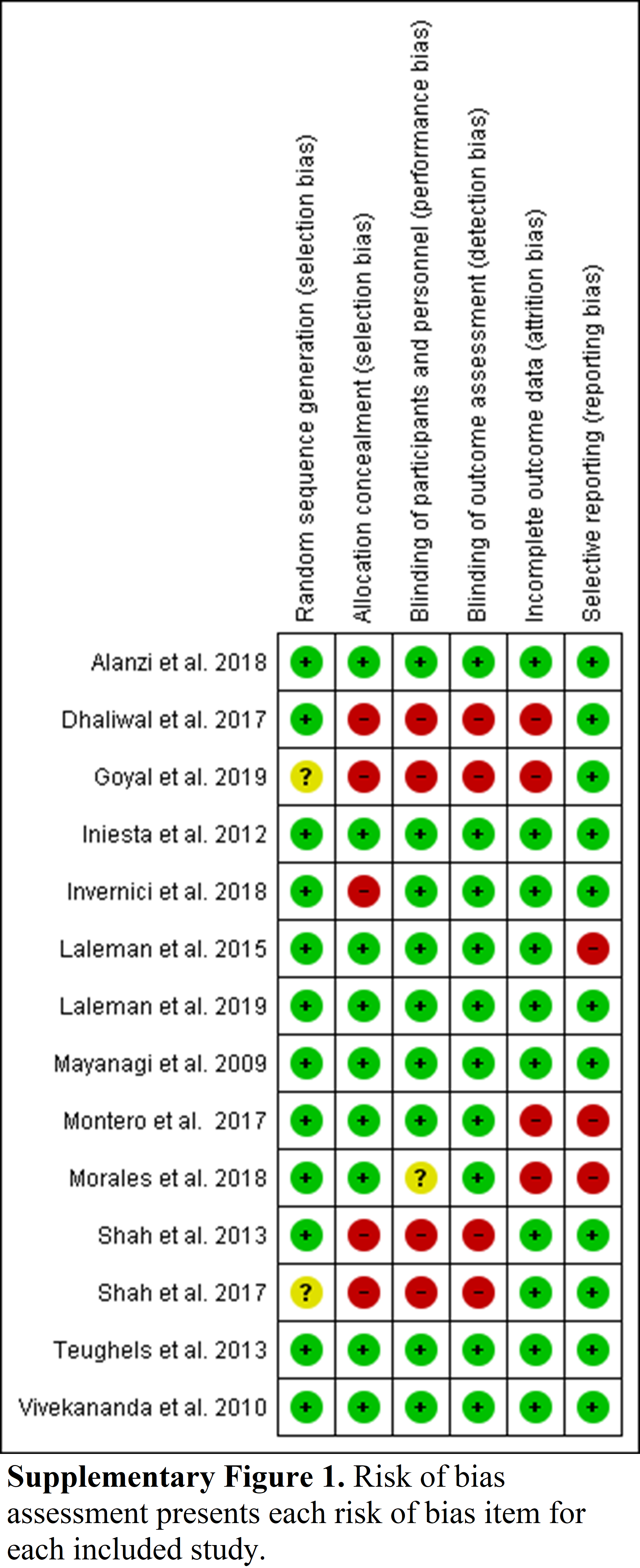

Supplement: Supplementary file 6 [file Image1.tif]

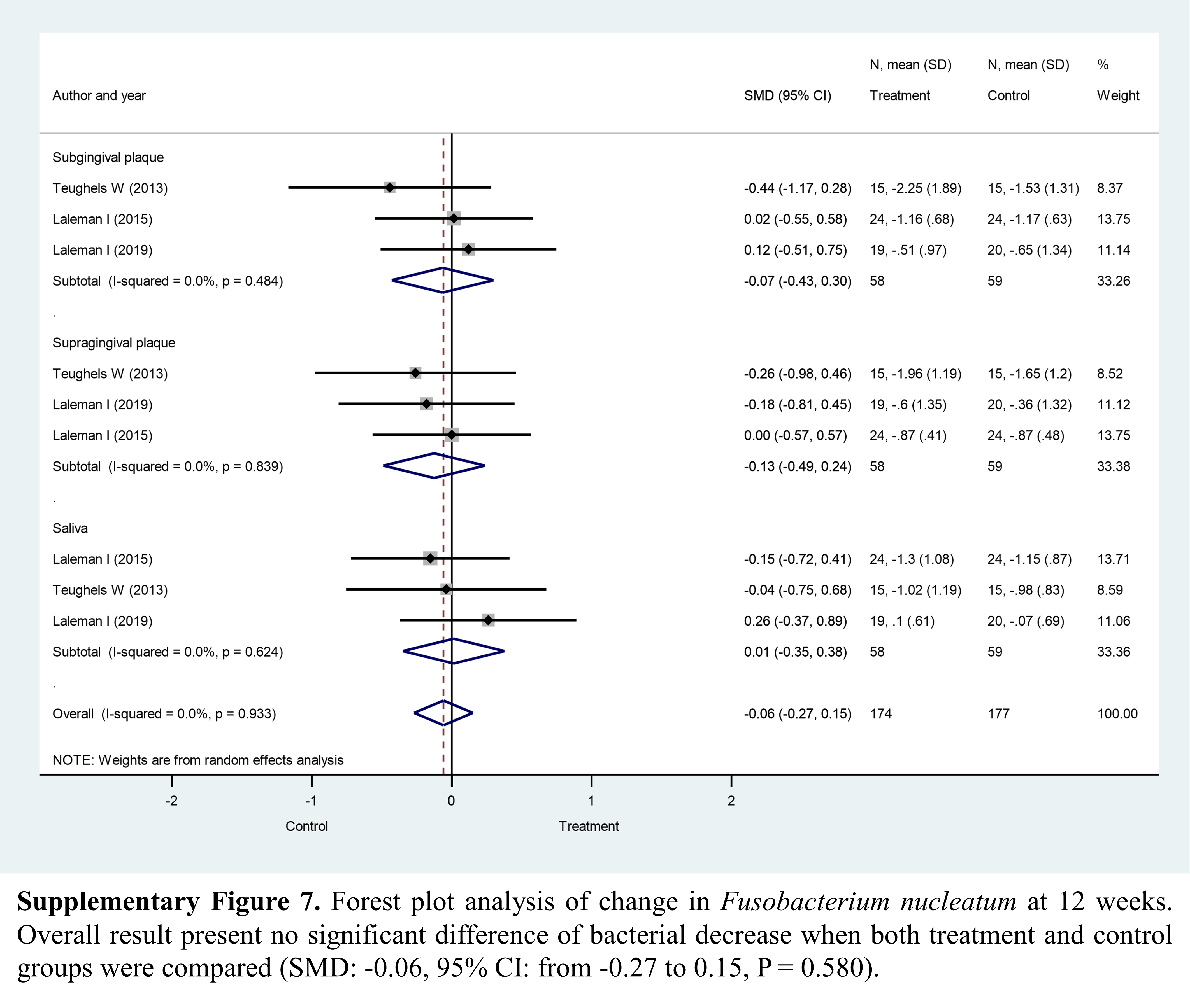

Supplement: Supplementary file 7 [file Image7.TIF]

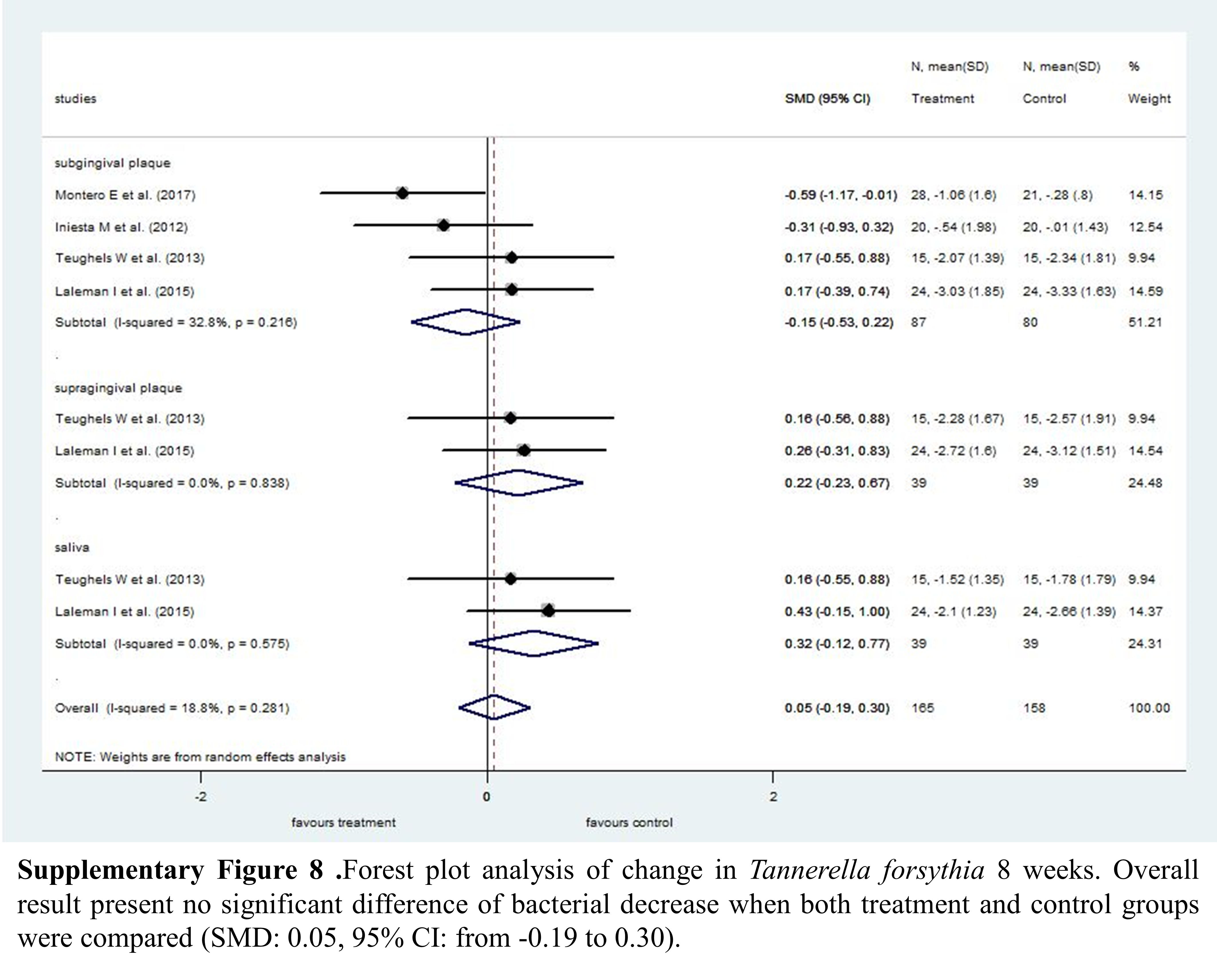

Supplement: Supplementary file 12 [file Image8.TIF]

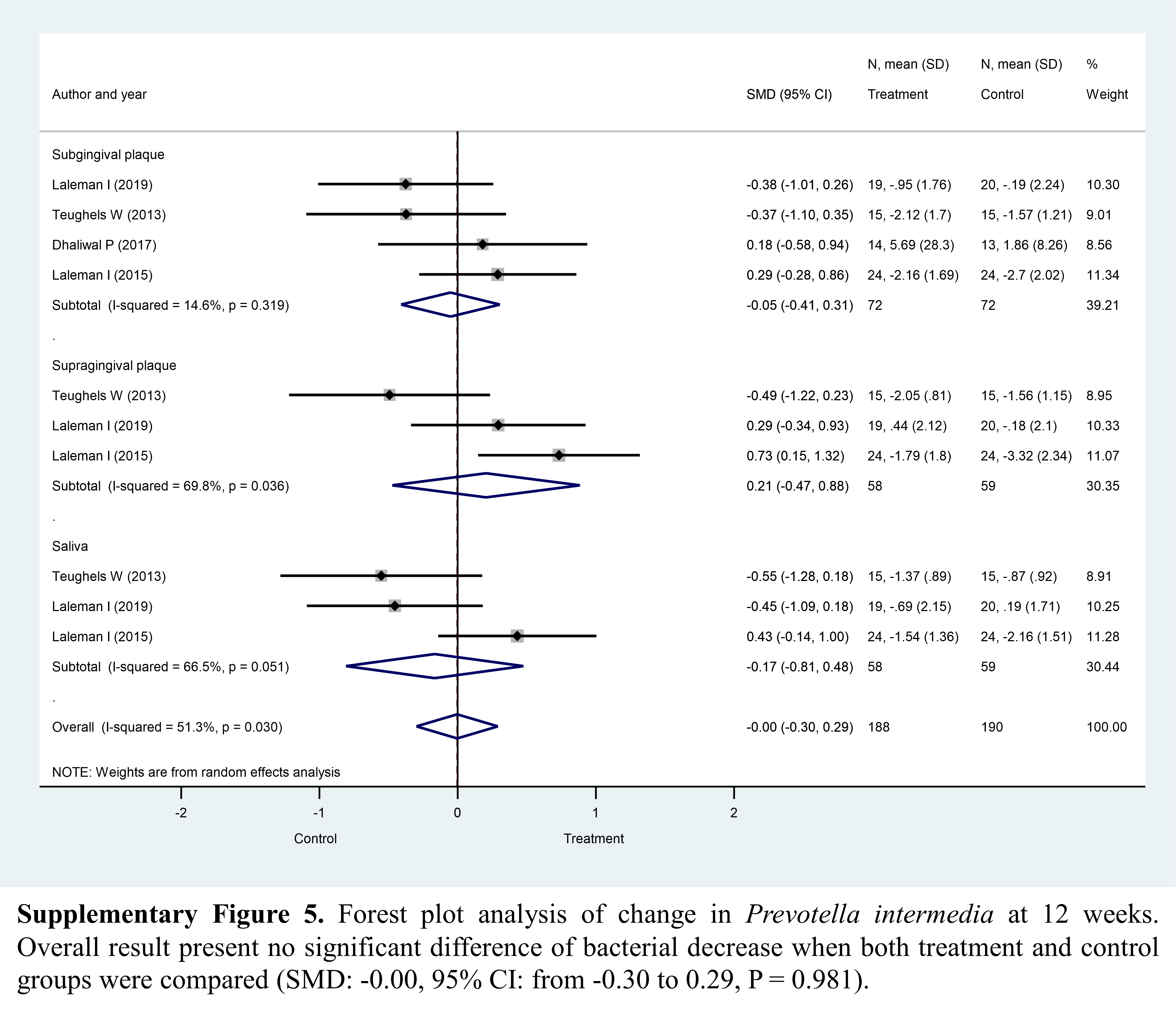

Supplement: Supplementary file 13 [file Image5.TIF]
