# Supplementary material for: Orally Administered Probiotics Decrease Aggregatibacter actinomycetemcomitans but Not Other Periodontal Pathogenic Bacteria Counts in the Oral Cavity: A Systematic Review and Meta-Analysis
Source: Front Pharmacol. 2021 Aug 6;12:682656. doi: 10.3389/fphar.2021.682656 (PMC8383782; doi:10.3389/fphar.2021.682656)
Supplement: Supplementary file 8 [file Table2.DOCX]

**Supplementary Table 2.** Search strategy.

| 1. Search query in **Pubmed**: | probiotic and ("periodontal disease" or periodontitis or gingivitis or plaque or saliva)  filter: human  ("probiotics"[MeSH Terms] OR "probiotics"[All Fields] OR "probiotic"[All Fields]) AND ("periodontal disease"[All Fields] OR ("periodontitis"[MeSH Terms] OR "periodontitis"[All Fields]) OR ("gingivitis"[MeSH Terms] OR "gingivitis"[All Fields]) OR ("plaque, amyloid"[MeSH Terms] OR ("plaque"[All Fields] AND "amyloid"[All Fields]) OR "amyloid plaque"[All Fields] OR "plaque"[All Fields] OR "dental plaque"[MeSH Terms] OR ("dental"[All Fields] AND "plaque"[All Fields]) OR "dental plaque"[All Fields]) OR ("saliva"[MeSH Terms] OR "saliva"[All Fields])) AND "humans"[MeSH Terms]  Filter: human filter |
| --- | --- |
| 2. Search query in **Embase**: | ('probiotic'/exp OR 'probiotic') AND ('periodontal disease'/exp OR 'periodontal disease' OR 'periodontitis'/exp OR periodontitis OR 'gingivitis'/exp OR gingivitis OR 'plaque'/exp OR plaque OR 'saliva'/exp OR saliva) AND [humans]/lim |
| 3.Search query in **Cochrane**: | probiotic and ("periodontal disease" or periodontitis or gingivitis or plaque or saliva) |
| 4. Search query in **Web of Science**: | probiotic and ("periodontal disease" or periodontitis or gingivitis or plaque or saliva) |
