# Supplementary material for: Orally Administered Probiotics Decrease Aggregatibacter actinomycetemcomitans but Not Other Periodontal Pathogenic Bacteria Counts in the Oral Cavity: A Systematic Review and Meta-Analysis
Source: Front Pharmacol. 2021 Aug 6;12:682656. doi: 10.3389/fphar.2021.682656 (PMC8383782; doi:10.3389/fphar.2021.682656)
Supplement: Supplementary file 9 [file Table5.DOCX]

**Supplementary Table 5.** GRADE evidence.

| **Outcomes** | **Certainty assessment** | | | | | | | **Certainty** |
| --- | --- | --- | --- | --- | --- | --- | --- | --- |
|  | **№ of studies** | **Study design** | **Risk of bias** | **Inconsistency** | **Indirectness** | **Imprecision** | **Publication bias** |  |
| The amount of *A. actinomycetemcomitans*  At 4 week | 4 | randomised trials | serious^1^ | Serious^2^ | not serious | very serious^5^ | undetected | ⨁◯◯◯ VERY LOW |
| The amount of *A. actinomycetemcomitans*  At 8 week | 4 | randomised trials | serious^1^ | serious^2^ | not serious | very serious^5^ | undetected | ⨁◯◯◯ VERY LOW |
| The amount of *P. gingivalis* At 4 week | 5 | randomised trials | serious^1^ | very serious^3^ | not serious | very serious^5^ | undetected | ⨁◯◯◯ VERY LOW |
| The amount of *P. gingivalis* At 8 week | 5 | randomised trials | serious^1^ | very serious^3^ | not serious | very serious^5^ | undetected | ⨁◯◯◯ VERY LOW |
| The amount of *P. gingivalis* At 12 week | 3 | randomised trials | not serious | very serious^3^ | not serious | very serious^5^ | undetected | ⨁◯◯◯ VERY LOW |
| The amount of *P. intermedia* At 4 week | 4 | randomised trials | not serious | serious^2^ | not serious | very serious^5^ | undetected | ⨁◯◯◯ VERY LOW |
| The amount of *P. intermedia* At 8 week | 3 | randomised trials | not serious | serious^2^ | not serious | very serious^5^ | undetected | ⨁◯◯◯ VERY LOW |
| The amount of *P. intermedia* At 12 week | 4 | randomised trials | serious^1^ | serious^2^ | not serious | very serious^5^ | undetected | ⨁◯◯◯ VERY LOW |
| The amount of *F. nucleatum* At 4 week | 5 | randomised trials | not serious | serious^2^ | not serious | very serious^5^ | undetected | ⨁◯◯◯ VERY LOW |
| The amount of *F. nucleatum* At 8 week | 4 | randomised trials | not serious | serious^2^ | not serious | very serious^5^ | undetected | ⨁◯◯◯ VERY LOW |
| The amount of *F. nucleatum* At 12 week | 3 | randomised trials | not serious | very serious^3^ | not serious | very serious^5^ | undetected | ⨁◯◯◯ VERY LOW |
| The amount of *T. forsythia* At 4 week | 4 | randomised trials | not serious | serious^2^ | not serious | very serious^5^ | undetected | ⨁◯◯◯ VERY LOW |
| The amount of *T. forsythia* At 8 week | 4 | randomised trials | not serious | serious^2^ | not serious | very serious^5^ | undetected | ⨁◯◯◯ VERY LOW |

1: One articles was assessed as high risk bias (Open trial), 2: The inconsistency is substantial., 3: The inconsistency should be considerable, 4: small sample size, 5: small sample size and 95% CI overlaps no effect.
