# Supplementary material for: Orally Administered Probiotics Decrease Aggregatibacter actinomycetemcomitans but Not Other Periodontal Pathogenic Bacteria Counts in the Oral Cavity: A Systematic Review and Meta-Analysis
Source: Front Pharmacol. 2021 Aug 6;12:682656. doi: 10.3389/fphar.2021.682656 (PMC8383782; doi:10.3389/fphar.2021.682656)
Supplement: Supplementary file 10 [file Table3.DOCX]

**Supplementary Table 3.** Summary of characteristics of the studies included.

| **Study, year** | **Country** | **Participants** | | | **Pre-treatment** | **Probiotics Treatment** | | | **Control**  **group** | **Primary outcome** | |
| --- | --- | --- | --- | --- | --- | --- | --- | --- | --- | --- | --- |
|  |  | **Number** | **Age (year)** | **Type of periodontal disease** |  | **Strain(dose)** | **Form (instruction)** | **Duration** |  | **Periodonto-pathogen** | **Follow up time (wk)** |
| **Alanzi et al., 2018** | Kuwait | 101 | 13-15 | Mild to moderate gingival inflammation | None | *Lactobacillus reuteri*, *Lactobacillus rhamnosus* GG, *Bifidobacterium lactis* BB-12 (4.4x10^8^/ 1g in each strain) | Lozenges (suck the lozenges twice a day) | 4 weeks | Placebo lozenges | *Aa*, *Pg*, *Pi*, *Fn*, total bacteria | Baseline,  4-week |
| **Dhaliwal et al., 2017** | India | 27 | 20-55 | Moderate to severe chronic generalized periodontitis | Scaling and root planing | *Streptococcus faecalis* T-110 JPC (30 million CFU), *Clostridium butyricum* TO-A HIS (2 million CFU), [*Bacillus mesentericus*](https://en.wikipedia.org/wiki/Bacillus_mesentericus) TO-A JPC (1million CFU), *Lactobacillus sporogenes* IHS-(50 million CFU) | Lozenges (suck the lozenges) | 21 days | None | *Aa*, *Pg*, *Pi* | Baseline,  4-, 6-, 12- week |
| **Goyal et al., 2019** | India | 30 | 15-35 | Not severe periodontal disease | None | *Lactobacillus reuteri*, *Lactobacillus rhamnosus*, *Bifidobacterium longum*, *Bifidobacterium Bifidum* (0.1 billion cfu/ 1g in each strain) | Mouthwash (swish 15 ml probiotic mouthwash for 60 s before swallowing, twice daily after brushing and do not eat anything for ½ h after rinsing) | Not mention | None | *Pg* | Baseline, 24-week |
| **Iniesta et al., 2012** | Spain | 40 | 20-24 | Gingivitis | Tooth polishing (rubber cup and abrasive paste) | *Lactobacillus reuteri* DSM 17938, *Lactobacillus reuteri* ATCC PTA 5289 (2x10^8^ cfu/tab) | Tablets (chew one tablet per day) | 28 days | Placebo tablet | *Aa*, *Pg*, *Pi*, *Fn*, *Tf*, total bacteria, Lactobacillus spp., *P. micra*, *C. rectus*, Capno., *E. corrodens* | Baseline, 4-,8-week |
| **Invernici et al., 2018** | Brazil | 41 | ≥ 30 | Generalized chronic periodontitis | Supra- and subgingival SRP on all teeth with hand and ultrasonic instruments, OHI | *Bifidobacterium lactis* HN019 (10^9^ CFUs) | Lozenges (take one lozenges twice a day after waking up and before bedtime) | 30 days | Placebo lozenges | *Aa*, *Pg*, *Pi*, *Fn*, *Tf*, other bacteria | Baseline,  4-, 12-week |
| **Laleman et al., 2019** | Belgium | 39 | 34-83 | Moderate to severe chronic periodontitis | Supragingival and subgingival SRP on all teeth with hand and ultrasonic instruments, OHI | *Lactobacillus reuteri* DSM 17938, *Lactobacillus reuteri* ATCC PTA 5289 (2x10^8^ cfu/5 drops or lozenge) | Drops (applied with a syringe and blunt needle in all residual pockets), Lozenges (dissolve on the tongue twice a day after brushing) | 12 weeks | Placebo drop and lozenges | *Aa*, *Pg*, *Pi*, *Fn* | Baseline, 12-, 24-week |

| **Study, year** | **Country** | **Participants** | | | **Pre-treatment** | **Probiotics Treatment** | | | **Control**  **group** | **Primary outcome** | |
| --- | --- | --- | --- | --- | --- | --- | --- | --- | --- | --- | --- |
|  |  | **Number** | **Age (year)** | **Type of periodontal disease** |  | **Strain (dose)** | **Form (instruction)** | **Duration** |  | **Periodonto-pathogen** | **Follow up time (wk)** |
| **Laleman et al., 2015** | Turkey | 48 | 37-58 | Moderate to severe adult periodontitis | 0.1% CHX rinse for 2 min, SRP under 0.12% CHX irrigation with hand and ultrasonic scaler, mucosal surfaces and tongue were disinfected by CHX gel for 1 min | *Streptococcus oralis* KJ3, *Streptococcus unberis* KJ2, *Streptococcus rattus* JH145 (10^8^ cfu/strain/tablet) | Tablets (dissolve on the tongue twice a day after brushing) | 3 months | Placebo tablets | *Pg*, *Pi*, *Fn*, *Tf* | Baseline, 4-, 8-, 12-, 24-week |
| **Mayana-gi et al., 2009** | Japan | 66 | 32-61 | Not severe periodontitis | None | *Lactobacillus salivarius* WB21 (6.7x10^8^ CFU/tab) | Tablets (place one tablet in the mouth and allow it to dissolve without chewing, 3 times a day) | 8 weeks | Placebo tablets | *Aa*, *Pg*, *Pi*, *Tf*, | Baseline,  4-, 8-week |
| **Montero et al., 2017** | Spain | 52 | 18-55 | Gingivitis | Supragingival and Subgingival SRP, OHI | *Lactobacillus plantarum* CECT 7481(AB15), *Lactobacillus brevis* CECT7480 (AB38), *Pediococcus acidilactici* CECT8633(AB30) (10^3^ CFUs/strain) | Tablet (chewing, twice a day, morning and night after OHI procedure) | 6 weeks | Placebo tablets | *Aa*, *Pg*, *Fn*, *Tf*, *C. rectus* | Baseline, 6- week |
| **Morales et al., 2018** | Chile | 47 | ≥ 35 | Chronic periodontitis | Supragingival and Subgingival SRP on all teeth with hand and ultrasonic instruments, OHI | *Lactobacillus rhamnosus* SP1  (2x10^7^ CFU/day) | Sachet (dissolve 1 sachet in 150 ml water and ingest it once a day after brushing teeth) | 3 months | Placebo sachet | *Aa*, *Pg*, *Tf*, | Baseline, 36-week |
| **Shah et al., 2017** | India | 18 | 14-35 | Aggressive periodontitis | Scaling and root planing | *Lactobacillus brevis* CD2  (10^9^ CFU/lozenge) | Lozenges (place the lozenges in the oral cavity for a few minutes, allowing them to dissolve and a doxycycline tablet once every day) | 14 days | A doxycycline tablet | *Aa* | Baseline, 2-, 8-, 20-week |
| **Shah et al., 2013** | India | 30 | 14-35 | Aggressive periodontitis | SRP on all teeth with hand and ultrasonic instruments | *Lactobacillus brevis* (10^8^ CFU/gram) | Lozenges (place the lozenges in the oral cavity for a few minutes, allowing them to dissolve and a doxycycline tablet once every day) | 14 days | A doxycycline tablet | *Aa* | Baseline, 2-, 8-week |
| **Teughels et al., 2013** | Turkey | 30 | ≥ 35 | moderate to severe generalized periodontitis | 0.1%CHX rinse for 2 min, SRP under 0.12% CHX irrigation with hand and ultrasonic scaler, mucosal surfaces were disinfected by CHX on a swap, OHI | *Lactobacillus reuteri* DSM 17938, ATCC PTA 5289 (10^8^ cfu/strain) | Lozenges (suck one lozenge twice a day in the morning and at night after tooth brushing) | 12 weeks | Placebo lozenges | *Aa*, *Pg*, *Pi*, *Fn*, *Tf*, other bacteria | Baseline, 3-, 6-, 9-, 12- week |
| **Vivekananda et al., 2010** | Not mention | 30 | 34-50 | Chronic periodontitis | SRP 2 quadrants with ultrasonic and hand instruments, OHI | *Lactobacillus reuteri* DSM 17938, ATCC PTA 5289 [10^8^ cfu/strain] | Lozenges (suck one lozenges in the morning and at night, after brushing teeth) | Day 21-42 | Placebo lozenges | *Aa*, *Pg*, *Pi* | Baseline, 6-week |

*Pg*: *Porphyromonas gingivalis*, *Pi*: *Prevotella intermedia*, *Fn*: *Fusobacterium nucleatum*, *Tf*: *Tanellera forsythia*, *Aa*: *Aggregatibacter actinomycetemcomitans*, *Td*: *Treponema denticola*.
