# Supplementary material for: Orally Administered Probiotics Decrease Aggregatibacter actinomycetemcomitans but Not Other Periodontal Pathogenic Bacteria Counts in the Oral Cavity: A Systematic Review and Meta-Analysis
Source: Front Pharmacol. 2021 Aug 6;12:682656. doi: 10.3389/fphar.2021.682656 (PMC8383782; doi:10.3389/fphar.2021.682656)
Supplement: Supplementary file 11 [file Table4.DOCX]

**Supplementary Table 4.** Detailed Risk of Bias assessment of included studies.

**First author and title:** Alanzi, 2018  
*Effect of Lactobacillus rhamnosus and Bifidobacterium lactis on gingival health,*

*dental plaque, and periodontopathogens in adolescents: a randomised placebocontrolled*

*clinical trial*

| **Bias** | **Authors judgment** | **Support for judgment** |
| --- | --- | --- |
| **Random sequence generation (selection bias)** | Low risk | Quote: "...The simple randomisation procedure to study and control groups was performed through the generation of computerised random numbers using Excel 2007(Microsoft, Redmond, WA, USA)." |
| **Allocation concealment (selection bias)** | Low risk | Quote: "...All children, teachers, and researchers were blinded to the type of lozenges assigned to them. Lozenges were in colour-coded plastic bottles, and the code was kept in secret by the company..." |
| **Blinding of participants and personnel (performance bias)** | Low risk | Quote: "...All children, teachers, and researchers were blinded to the type of lozenges assigned to them. Lozenges were in colour-coded plastic bottles, and the code was kept in secret by the company. These codes were disclosed after the statistical analysis procedure had been conducted." |
| **Blinding of outcome assessment (detection bias)** | Low risk | Quote: "...All children, teachers, and researchers were blinded to the type of lozenges assigned to them. Lozenges were in colour-coded plastic bottles, and the code was kept in secret by the company. These codes were disclosed after the statistical analysis procedure had been conducted." |
| **Incomplete outcome data (attrition bias)** | Low risk | Comment: Although there are withdrawal subjects (2 persons from 54 persons in test group, 5 persons from 54 persons in control group), the data were analyzed by appropriate methods and not seems to affect the result. |
| **Selective reporting (reporting bias)** | Low risk | Comment: All pre-specified outcomes of interest are reported. |

**First author and title:** Dhaliwal, 2017  
*Clinical and Microbiological Investigation of the Effects of Probiotics Combined with*

*Scaling and Root Planing in the Management of Chronic Periodontitis: A Randomized,*

*Controlled Study*

| **Bias** | **Authors judgment** | **Support for judgment** |
| --- | --- | --- |
| **Random sequence generation (selection bias)** | Low risk | Quote: "Patients were randomly assigned (coin-toss method) to 2 groups... |
| **Allocation concealment (selection bias)** | High risk | Comment: No allocation concealment is explained. Additionally, control group didn't receive any placebo lozenges. Therefore, the allocation concealment wasn't done. |
| **Blinding of participants and personnel (performance bias)** | High risk | Comment: No performance blinding of both participants and personnel are mentioned or described |
| **Blinding of outcome assessment (detection bias)** | High risk | Comment: No performance blinding of examiner is mentioned or described. |
| **Incomplete outcome data (attrition bias)** | High risk | Comment: Three enrolled participants lost follow-up, however, those data were excluded and the available data were statitically analyzed. |
| **Selective reporting (reporting bias)** | Low risk | Comment: All pre-specified outcomes of interest are reported. |

**First author and title:** Goyal, 2019  
*Effects of amine fluoride and probiotic mouthwash on levels of Porphyromonas gingivalis in*

*orthodontic patients: A randomized controlled trial*

| **Bias** | **Authors judgment** | **Support for judgment** |
| --- | --- | --- |
| **Random sequence generation (selection bias)** | Unclear risk | Quote: "Randomisation was performed and patients were equally divided into three groups..."  Comment: The detail of randomization wasn't explained in the article. |
| **Allocation concealment (selection bias)** | High risk | Comment: The allocation concealment wasn’t stated. |
| **Blinding of participants and personnel (performance bias)** | High risk | Comment: The sample groups received the different treatments and the treatments could be known by the instruction of use. |
| **Blinding of outcome assessment (detection bias)** | High risk | Comment: No blinding was indicated. |
| **Incomplete outcome data (attrition bias)** | High risk | Comment: The droup out patients weren't explanied. |
| **Selective reporting (reporting bias)** | Low risk | Comment: All pre-specified outcomes of interest are reported. |

| **First author and title:** Hallstrom, 2013 *Effect of probiotic lozenges on inflammatory reactions and oral biofilm*  *during experimental gingivitis*   \| **Bias** \| **Authors judgment** \| **Support for judgment** \| \| --- \| --- \| --- \| \| **Random sequence generation (selection bias)** \| Low risk \| Quote: "...The randomization was carried out with the aid of the computerized Excel randomization tool..." \| \| **Allocation concealment (selection bias)** \| Low risk \| Quote. "...All study subjects, the laboratory technician and involved clinicians were blinded for the group allocation..." \| \| **Blinding of participants and personnel (performance bias)** \| Low risk \| Quote. "...All study subjects, the laboratory technician and involved clinicians were blinded for the group allocation...". \| \| **Blinding of outcome assessment (detection bias)** \| Low risk \| Quote. "...All study subjects, the laboratory technician and involved clinicians were blinded for the group allocation..." \| \| **Incomplete outcome data (attrition bias)** \| Low risk \| Comment: There is no lost follow-up participant. \| \| **Selective reporting (reporting bias)** \| Low risk \| Comment: All pre-specified outcomes of interest are reported. \|   **First author and title:** Iniesta, 2012 *Probiotic effects of orally administered Lactobacillus reuteri-containing tablets on the subgingival and salivary microbiota in patients with gingivitis. A randomized clinical trial*   \| **Bias** \| **Authors judgment** \| **Support for judgment** \| \| --- \| --- \| --- \| \| **Random sequence generation (selection bias)** \| Low risk \| Quote: “Subjects were randomly assigned following a computer-generated randomization list” \| \| **Allocation concealment (selection bias)** \| Low risk \| Quote: “The test and placebo tablets were provided in identical packages by BioGaia AB (Stockholm, Sweden) and were identiﬁed with the study number. The codes were not broken until the end of the study.”  Comment: All of the subjects were received the same instruction and baseline evaluation. \| \| **Blinding of participants and personnel (performance bias)** \| Low risk \| Comment: Personnel blinding was not described. \| \| **Blinding of outcome assessment (detection bias)** \| Low risk \| Quote: “The study was initially designed as a double-blind (subject, evaluators), prospective, placebo-controlled, crossover randomized clinical trial...”  Comment: No blinding described. \| \| **Incomplete outcome data (attrition bias)** \| Low risk \| Comment: Two outcome in saliva samples lost and the author stated the reason. \| \| **Selective reporting (reporting bias)** \| Low risk \| Comment: All pre-specified outcomes of interest are reported. Although there were some results lost, it seems to be laboratory technical problem. \|   **First author and title:** Invernici, 2018 *Effects of Bifidobacterium probiotic on the treatment of chronic periodontitis: A randomized clinical trial* | | |
| --- | --- | --- | --- | --- | --- | --- | --- | --- | --- | --- | --- | --- | --- | --- | --- | --- | --- | --- | --- | --- | --- | --- | --- | --- | --- | --- | --- | --- | --- | --- | --- | --- | --- | --- | --- | --- | --- | --- | --- | --- | --- | --- | --- | --- |
| **Bias** | **Authors judgment** | **Support for judgment** |
| **Random sequence generation (selection bias)** | Low risk | Quote: "According to a random numeric table generated by computer software, the study coordinator (M.R.M.) allocated each patient to one of the following groups..." |
| **Allocation concealment (selection bias)** | High risk | Comment: The study coordinator who allocated the patient to the groups and identified the code was not blinded. |
| **Blinding of participants and personnel (performance bias)** | Low risk | Comment: Both participants and performer were blinded by using code. |
| **Blinding of outcome assessment (detection bias)** | Low risk | Quote: "The evaluations (pre-and post-intervention)were conducted by a single trained and calibrated examiner (M.S.M.S.), who was blinded to the experimental groups." |
| **Incomplete outcome data (attrition bias)** | Low risk | Comment: There is no lost follow-up participant. |
| **Selective reporting (reporting bias)** | Low risk | Comment: All pre-specified outcomes of interest are reported. |

| **First author and title:** Laleman, 2015  *The effect of a streptococci containing probiotic in periodontal therapy: a randomized controlled trial* | | |
| --- | --- | --- |
| **Bias** | **Authors judgment** | **Support for judgment** |
| **Random sequence generation (selection bias)** | Low risk | Quote: ”Randomization of the patients was done by block randomization (version 2.7.3; StatsDirect).” |
| **Allocation concealment (selection bias)** | Low risk | Quote: “the probiotic and placebo tablets were identical in shape, texture, taste and compostion.”  Comment: All of the subjects were received the same pretreatment, instruction and baseline evaluation. |
| **Blinding of participants and personnel (performance bias)** | Low risk | Quote: “Except for the study coordinator, all patients and study personnel were blinded to the study group allocation. |
| **Blinding of outcome assessment (detection bias)** | Low risk | Quote: “Except for the study coordinator, all patients and study personnel were blinded to the study group allocation.” |
| **Incomplete outcome data (attrition bias)** | Low risk | Quote: “ No data are shown for *Aggregatibacter actinomycetemcomitans* since the vast majority of the obtained data were below the detection limit, which made statistical analysis impossible”  Comment: The lost data was reported with reason. |
| **Bias** | **Authors judgment** | **Support for judgment** |
| **Selective reporting (reporting bias)** | Unclear risk | Comment: All pre-specified outcomes of interest are reported except the *A. actinomycetemcomitans* amount. |

| **First author and title:** Laleman, 2019  *A dual‐strain Lactobacilli reuteri probiotic improves the treatment of residual pockets: A randomized controlled clinical trial* | | |
| --- | --- | --- |
| **Bias** | **Authors judgment** | **Support for judgment** |
| **Random sequence generation (selection bias)** | Low risk | Quote: "... This was done based on a computer‐generated table (www.rando mizat ion.com) that linked each patient to one of the treatment groups." |
| **Allocation concealment (selection bias)** | Low risk | Quote: "The randomization of the study protocols was performed by a staff member who was not further involved in this study..." |
| **Blinding of participants and personnel (performance bias)** | Low risk | Quote: "...The similarity of the packaging, and the identical appearance, texture and taste of the study products (both the drops as the lozenges) made the double‐blinding of the researcher and patient possible..." |
| **Blinding of outcome assessment (detection bias)** | Low risk | Comment: Only one experienced periodontist who were blinded from the allocation performed on clinical treatment and sample collection. |
| **Incomplete outcome data (attrition bias)** | Low risk | Comment: There were lost follow-up patients. However, the proper statical theory were used for data analysis. |
| **Selective reporting (reporting bias)** | Low risk | Comment: All pre-specified outcomes of interest are reported. |

**First author and title:** Mayanagi, 2009

*Probiotic effects of orally administered Lactobacillus salivarius WB21-containing tablets*

*on periodontopathic bacteria: a double-blinded, placebo controlled, randomized clinical trial*

| **Bias** | **Authors judgment** | **Support for judgment** |
| --- | --- | --- |
| **Random sequence generation (selection bias)** | Low risk | Quote: "There were no significant differences between the WB21 and placebo groups randomized according to gender, age, smoking habits, and clinical features"  Comment: The randomization probably was done. However, there is no detail of randomization method. |
| **Allocation concealment (selection bias)** | Low risk | Quote: "The subjects were randomized into two groups to receive test (WB21 group) and control (placebo group) treatments after the BL examination..."  Comment: All of the subjects were received the same pretreatment, instruction and baseline evaluation. |
| **Blinding of participants and personnel (performance bias)** | Low risk | Quote: "A randomized, double-blinded, placebo-controlled study design with two parallel groups was used in this study..."  Comment: The blinding probably was done. |
| **Blinding of outcome assessment (detection bias)** | Low risk | Quote: "A randomized, double-blinded, placebo-controlled study design with two parallel groups was used in this study..."  Comment: The author did not indicate or explain about the blinding assessor. |
| **Bias** | **Authors judgment** | **Support for judgment** |
| **Incomplete outcome data (attrition bias)** | Low risk | Comment: One subject lost in control group and the author explained the reason clearly. |
| **Selective reporting (reporting bias)** | Low risk | Comment: All pre-specified outcomes of interest are reported. |

**First author and title:** Montero, 2017

*Clinical and microbiological effects of the adjunctive use of probiotics in the treatment of gingivitis: A randomized controlled clinical trial*

| **Bias** | **Authors judgment** | **Support for judgment** |
| --- | --- | --- |
| **Random sequence generation (selection bias)** | Low risk | Quote: “Subjects were randomly assigned by blocks using a computer- generated list to one of the following two regimen…”  Comment: The gender ratio and age are quite the same in test and control group. |
| **Allocation concealment (selection bias)** | Low risk | Quote: “All tablets, as well as the cases containing them, were identical and were coded according to the computer- generated randomization list, which was only revealed at the end of the study.”  Comment : All of the subjects were received the same pretreatment, instruction and baseline evaluation. |
| **Blinding of participants and personnel (performance bias)** | Low risk | Quote: “The study was designed as a randomized, double- blinded, placebo- controlled, parallel- group clinical trial…”  Comment: No blinding described. |
| **Blinding of outcome assessment (detection bias)** | Low risk | Comment: No details about microbiological examiner. |
| **Bias** | **Authors judgment** | **Support for judgment** |
| **Incomplete outcome data (attrition bias)** | High risk | Comment: Some samples lost in microbiological outcome and there was no explanation. |
| **Selective reporting (reporting bias)** | High risk | Comment: Although all pre-specified outcomes of interest are reported, some microbiological data lost without any explanation. |

**First author and title:** Morales, 2018

*Microbiological and clinical effects of probiotics and antibiotics on nonsurgical treatment of chronic periodontitis: a randomized placebo controlled trial with 9-month follow-up*

| **Bias** | **Authors judgment** | **Support for judgment** |
| --- | --- | --- |
| **Random sequence generation (selection bias)** | Low risk | Quote: "According to gender, age, and smoking status, eligible individuals were randomly allocated to groups after the basal examination, using a computer-generated randomization table (Jorge Gamonal)..."  Comment: The patient's age, gender are quite similar in each group. Thus the random sequence generation probably was done. |
| **Allocation concealment (selection bias)** | Low risk | Quote: "Allocation concealment was prepared using sequentially numbered, opaque sealed envelopes." |
| **Blinding of participants and personnel (performance bias)** | Unclear risk | Comment: The study coordinator collected subgingival sample was not blinded and used a computer program generating randomization. The performance bias might happen. |
| **Blinding of outcome assessment (detection bias)** | Low risk | Comment: Microbiological procedures were performed by one expert who was not involve in other process in the study. |
| **Bias** | **Authors judgment** | **Support for judgment** |
| **Incomplete outcome data (attrition bias)** | High risk | Comment: The article reported no participants had lost in the study. However, the study did not report the percentages of *T. forsythia* and *A. actinomycetemcomitans* as stated in the method part. |
| **Selective reporting (reporting bias)** | High risk | Comment: The percentages of *T. forsythia* and *A. actinomycetemcomitans* were not reported at measurement point time. Also the percentage *P. gingivalis* and prevalence of these bacteria at 3 and 6 months were not shown in the article. |

**First author and title:** Shah, 2013

*Evaluation of the Effect of Probiotic (Inersan®) Alone, Combination of Probiotic with Doxycycline and Doxycycline Alone on Aggressive Periodontitis – A Clinical and Microbiological Study*

| **Bias** | **Authors judgment** | **Support for judgment** |
| --- | --- | --- |
| **Random sequence generation (selection bias)** | Low risk | Quote: "By block randomization, a total of thirty patients of both genders (fourteen males and sixteen females; mean age 24.83 ± 4.42 years, age range 14-35 years) were included..."  Comment: There is no description about randomization, the randomization probably was done. |
| **Allocation concealment (selection bias)** | High risk | Comment: Allocation concealment was not described and there was no intervention concealment. |
| **Blinding of participants and personnel (performance bias)** | High risk | Quote: "On the same day, medications were given to the patients for fourteen days according to the groups which they belonged to..."  Comment: Participants in probiotic plus antibiotic group and antibiotic group received different medical instruction. Therefore, the participants might know their intervention. The examiner blinding was not stated. |
| **Blinding of outcome assessment (detection bias)** | High risk | Comment: The study did not indicate the examiner and explain the blinding examiner.  Comment: No blinding described. |
| **Bias** | **Authors judgment** | **Support for judgment** |
| **Incomplete outcome data (attrition bias)** | Low risk | Comment: All s were reported following the study method. |
| **Selective reporting (reporting bias)** | Low risk | Comment: All pre-specified outcomes of interest are reported. |

**First author and title:** Shah, 2017

*Long-term effect of Lactobacillus brevis CD2 (Inersan®) and/or doxycycline in aggressive periodontitis*

| **Bias** | **Authors judgment** | **Support for judgment** |
| --- | --- | --- |
| **Random sequence generation (selection bias)** | Unclear risk | Quote: " Methods are described in full by Shah(14).”  Comment: The same risk assessment as in Shah, 2013 is applied |
| **Allocation concealment (selection bias)** | High risk | Quote: " Methods are described in full by Shah(14).”  Comment: The same risk assessment as in Shah, 2013 is applied |
| **Blinding of participants and personnel (performance bias)** | High risk | Quote: " Methods are described in full by Shah(14).”  Comment: The same risk assessment as in Shah, 2013 is applied |
| **Blinding of outcome assessment (detection bias)** | High risk | Quote: " Methods are described in full by Shah(14).”  Comment: The same risk assessment as in Shah, 2013 is applied |
| **Incomplete outcome data (attrition bias)** | Low risk | Comment: All s were reported following the study method. |
| **Selective reporting (reporting bias)** | Low risk | Comment: All pre-specified outcomes of interest are reported. |

| **Article’s first author and title:** Teughels, 2013  *Clinical and microbiological effects of Lactobacillus reuteri probiotics in the treatment of chronic periodontitis: a randomized placebo-controlled study* | | |
| --- | --- | --- |
| **Bias** | **Authors judgment** | **Support for judgment** |
| **Random sequence generation (selection bias)** | Low risk | Quote: “the participants were randomized by the study coordinator(MCH) over the two treatment groups …the two different treatment groups was done by block randomization (version 2.7.3; StatsDirect). ”  Comment: The coordinator probably use the program for randomization. |
| **Allocation concealment (selection bias)** | Low risk | Quote: “Both the probiotic and placebo lozenges could not be discriminated from each other by shape, texture or taste.”  Comment: All of the subjects were received the same pretreatment, instruction and baseline evaluation. |
| **Blinding of participants and personnel (performance bias)** | Low risk | Quote: “Except for the study co-ordinator, all study personnel and patients were blinded to the study group assignment..”  Comment: The blinding probably was done. |
| **Blinding of outcome assessment (detection bias)** | Low risk | Comment: The microbiological examiner was not described. |
| **Bias** | **Authors judgment** | **Support for judgment** |
| **Incomplete outcome data (attrition bias)** | Low risk | Comment: There is no lost follow-up or discontinued use of probiotics patients. |
| **Selective reporting (reporting bias)** | Low risk | Comment: All pre-specified outcomes of interest are reported. |

| **Article’s first author and title:** Vivekananda, 2010  *Effect of the probiotic Lactobacilli reuteri (Prodentis) in the management of periodontal disease: a preliminary randomized clinical trial* | | |
| --- | --- | --- |
| **Bias** | **Authors judgment** | **Support for judgment** |
| **Random sequence generation (selection bias)** | Low risk | Quote: “All subjects were assigned to one of the two groups (Prodentis group or placebo group) by using a randomization  table ”  Comment: The randomization probably has been done. |
| **Allocation concealment (selection bias)** | Low risk | Quote: “The randomization was concealed by using sequentially numbered, identical-appearing containers of probiotic or placebo tablets.” |
| **Blinding of participants and personnel (performance bias)** | Low risk | Quote: “To maintain full blinding of the results, the randomization code was held by one of the authors remotely from all assessments and was not broken until all data had been collected and all analyses had been performed.”  Comment: The blinding probably was done. |
| **Blinding of outcome assessment (detection bias)** | Low risk | “…the randomization code was held by one of the authors remotely from all assessments…” |
| **Incomplete outcome data (attrition bias)** | Low risk | Comment: There is no lost follow-up or discontinued use of probiotics patients |
| **Bias** | **Authors judgment** | **Support for judgment** |
| **Selective reporting (reporting bias)** | Low risk | Comment: All pre-specified outcomes of interest are reported. |
